# Influence of anthropogenic emissions and boundary conditions on multi-model simulations of major air pollutants over Europe and North America in the framework of AQMEII3

**DOI:** 10.5194/acp-18-8929-2018

**Published:** 2018

**Authors:** Ulas Im, Jesper Heile Christensen, Camilla Geels, Kaj Mantzius Hansen, Jørgen Brandt, Efisio Solazzo, Ummugulsum Alyuz, Alessandra Balzarini, Rocio Baro, Roberto Bellasio, Roberto Bianconi, Johannes Bieser, Augustin Colette, Gabriele Curci, Aidan Farrow, Johannes Flemming, Andrea Fraser, Pedro Jimenez-Guerrero, Nutthida Kitwiroon, Peng Liu, Uarporn Nopmongcol, Laura Palacios-Peña, Guido Pirovano, Luca Pozzoli, Marje Prank, Rebecca Rose, Ranjeet Sokhi, Paolo Tuccella, Alper Unal, Marta G. Vivanco, Greg Yarwood, Christian Hogrefe, Stefano Galmarini

**Affiliations:** 1 Aarhus University, Department of Environmental Science, Frederiksborgvej 399, Roskilde, Denmark; 2 European Commission, Joint Research Centre (JRC), Ispra, Italy; 3 Eurasia Institute of Earth Sciences, Istanbul Technical University, Istanbul, Turkey; 4 Ricerca sul Sistema Energetico (RSE SpA), Milan, Italy; 5 University of Murcia, Department of Physics, Physics of the Earth, Campus de Espinardo, Facultad de Química, Murcia, Spain; 6 Enviroware srl, Concorezzo, Italy; 7 Institute of Coastal Research, Chemistry Transport Modelling Group, Helmholtz-Zentrum Geesthacht, Geesthacht, Germany; 8 INERIS, Institut National de l’Environnement Industriel et des Risques, Parc Alata, Verneuil-en-Halatte, France; 9 Dept. Physical and Chemical Sciences, University of L’Aquila, L’Aquila, Italy; 10 Center of Excellence CETEMPS, University of L’Aquila, L’Aquila, Italy; 11 Centre for Atmospheric and Instrumentation Research (CAIR), University of Hertfordshire, Hatfield, UK; 12 European Centre for Medium-Range Weather Forecasts (ECMWF), Reading, UK; 13 Ricardo Energy & Environment, Gemini Building, Fermi Avenue, Harwell, Oxon, UK; 14 Environmental Research Group, Kings’ College London, London, UK; 15 NRC Research Associate at Computational Exposure Division, National Exposure Research Laboratory, Office of Research and Development, United States Environmental Protection Agency, Research Triangle Park, NC, USA; 16 Ramboll Environ, 773 San Marin Drive, Suite 2115, Novato, CA, USA; 17 Finnish Meteorological Institute, Atmospheric Composition Research Unit, Helsinki, Finland; 18 Cornell University, Department of Earth and Atmospheric Sciences, Ithaca, NY, USA; 19 CIEMAT, Avda. Complutense 40, Madrid, Spain; 20 Computational Exposure Division, National Exposure Research Laboratory, Office of Research and Development, United States Environmental Protection Agency, Research Triangle Park, NC, USA; a now at: Section Environmental Meteorology, Division Customer Service, ZAMG e Zentralanstalt für Meteorologie und Geodynamik, Vienna, Austria

## Abstract

In the framework of the third phase of the Air Quality Model Evaluation International Initiative (AQMEII3), and as contribution to the second phase of the Hemispheric Transport of Air Pollution (HTAP2) activities for Europe and North America, the impacts of a 20 % decrease of global and regional anthropogenic emissions on surface air pollutant levels in 2010 are simulated by an international community of regional-scale air quality modeling groups, using different state-of-the-art chemistry and transport models (CTMs). The emission perturbations at the global level, as well as over the HTAP2-defined regions of Europe, North America and East Asia, are first simulated by the global Composition Integrated Forecasting System (C-IFS) model from European Centre for Medium-Range Weather Forecasts (ECMWF), which provides boundary conditions to the various regional CTMs participating in AQMEII3. On top of the perturbed boundary conditions, the regional CTMs used the same set of perturbed emissions within the regional domain for the different perturbation scenarios that introduce a 20 % reduction of anthropogenic emissions globally as well as over the HTAP2-defined regions of Europe, North America and East Asia.

Results show that the largest impacts over both domains are simulated in response to the global emission perturbation, mainly due to the impact of domestic emission reductions. The responses of NO_2_, SO_2_ and PM concentrations to a 20 % anthropogenic emission reduction are almost linear (~ 20 % decrease) within the global perturbation scenario with, however, large differences in the geographical distribution of the effect. NO_2_, CO and SO_2_ levels are strongly affected over the emission hot spots. O_3_ levels generally decrease in all scenarios by up to ~ 1 % over Europe, with increases over the hot spot regions, in particular in the Benelux region, by an increase up to ~ 6 % due to the reduced effect of NO_x_ titration. O_3_ daily maximum of 8 h running average decreases in all scenarios over Europe, by up to ~ 1 %. Over the North American domain, the central-to-eastern part and the western coast of the US experience the largest response to emission perturbations. Similar but slightly smaller responses are found when domestic emissions are reduced. The impact of intercontinental transport is relatively small over both domains, however, still noticeable particularly close to the boundaries. The impact is noticeable up to a few percent, for the western parts of the North American domain in response to the emission reductions over East Asia. O_3_ daily maximum of 8 h running average decreases in all scenarios over north Europe by up to ~ 5 %. Much larger reductions are calculated over North America compared to Europe.

In addition, values of the Response to Extra-Regional Emission Reductions (RERER) metric have been calculated in order to quantify the differences in the strengths of nonlocal source contributions to different species among the different models. We found large RERER values for O_3_ (~ 0.8) over both Europe and North America, indicating a large contribution from non-local sources, while for other pollutants including particles, low RERER values reflect a predominant control by local sources. A distinct seasonal variation in the local vs. non-local contributions has been found for both O_3_ and PM_2.5_, particularly reflecting the springtime long-range transport to both continents.

## 1 Introduction

Regional air quality modeling has considerably developed during recent decades, driven by increased concern regarding the impact of air pollution on human health and ecosystems. Numerous air quality models have been developed by research groups worldwide and are being widely used for developing and testing emission control policies. Regional atmospheric chemistry and transport models (CTMs) are widely used to assess the past, present and future levels of air pollutants from continental to regional scales. There are different sources of uncertainties in models such as emissions, meteorology, boundary conditions and chemical schemes that should be taken into account when analyzing results. These uncertainties become more critical when these models are used for regulatory applications such as impacts of emission reductions. Multi-model ensembles can help in reducing this uncertainty and provide a better estimate of impacts under different scenarios (Solazzo et al., 2013; Galmarini et al., 2013; Kioutsioukis et al., 2016).

Numerous observational and modeling studies show that long-range transport of pollutants degrades air quality over remote continents (e.g., Wilkening et al., 2000; Holloway et al., 2003; Akimoto, 2003; Fiore et al., 2009). Although the influence of foreign emissions on continental scales is seen most frequently in the free troposphere, surface levels can also be affected, in particular over locations that generally receive clean air masses (e.g., Li et al., 2002). For example, dust storms and biomass burning can influence the tropospheric composition on a hemispheric scale (e.g., Husar et al., 2001; Jaffe et al., 2004). Reducing air pollution levels in surface air would improve public health as exposure to these atmospheric constituents aggravates respiratory illness and leads to premature mortality (World Health Organization, 2013; Im et al., 2018; Liang et al., 2018). However, attributing pollution to specific source regions is complicated due to the different processes influencing intercontinental transport and a large hemispheric background, and the dominance of local emissions in contributing to high levels of particular pollutants, such as ozone (O_3_) (e.g., Fiore et al., 2009). Given these difficulties, estimates of source-receptor relationships rely heavily on models.

Stjern et al. (2016), using 10 models participating in the second Hemispheric Transport of Air Pollution (HTAP2) activity, showed that a 20 % reduction of global anthropogenic emissions leads to significant changes regionally. They found that for North America (NA), black carbon emissions controls in East Asia are more important than domestic mitigation. In the framework of the HTAP2 activity, the UN (2007) showed that a 20 % reduction of North American NO_x_ emissions leads to a 0.22 ppb decrease in O_3_ levels over Europe (EU), while a 20 % decrease in East Asian NO_x_ emissions leads to a decrease of North American surface O_3_ levels by 0.12 ppb. The impacts of these emission changes on the O_3_ levels in the source regions are much higher. The impact of lateral boundary conditions (LBCs) on concentration fields simulated by regional-scale air quality models can also be quite significant (Jimédnez et al., 2007; Mathur, 2008; Rudich et al., 2008; Song et al., 2008; Andersson et al., 2015; Giordano et al., 2015; Hogrefe et al., 2018; Solazzo et al., 2017a). Recently, Giordano et al. (2015) showed that the regional models can be very sensitive to the boundary conditions provided by the global models. Tang et al. (2007) showed that the simulated surface levels over polluted areas are usually not as sensitive to the variation of LBCs but are more sensitive to the magnitude of their background concentrations. Jonson et al. (2018), in the framework of the HTAP2 activity, showed that for ozone the contributions from the rest of the world are larger than the effects from European emissions alone, with the largest contributions from North America and East Asia. The majority of these studies that address impact of emissions on regional and intercontinental transport employ global models on coarse spatial resolution or focus on just a few species, such as O_3_ or carbon monoxide (CO). On the other hand, studies using regional chemistry and transport models at finer spatial resolutions mostly focus on subregional scales (e.g., Im and Kanakidou, 2012; Huszar et al., 2016). Therefore, studies addressing multi-pollutant, source-receptor relationships on intercontinental and regional scales can provide valuable information on the impact of domestic and foreign emissions on regional air pollution levels. Multimodel ensembles operating on fine spatial resolutions can increase accuracy and provide an estimate of uncertainty.

The Air Quality Model Evaluation International Initiative (AQMEII), coordinated jointly by European Commission Joint Research Centre (EC-JRC) and the U.S. Environmental Protection Agency (EPA) has brought together regional chemistry and transport modeling groups from Europe and North America since 2008 (Rao et al., 2012; Solazzo et al., 2012a, b; Im et al., 2015a, b). AQMEII is now running its third phase as a regional subproject of the larger HTAP, which in turn is a task force of Long Range Transport of Air Pollution (LTRAP) program of the United Nations Economic Commission for Europe (UNECE) (Galmarini et al., 2017). The aim of the study is to assess the impact of global and HTAP2-defined regional anthropogenic emission reductions of 20 % in Europe, North America and East Asia on major air pollutant levels over Europe and North America using a multi-model ensemble approach. The study will also investigate the local vs. non-local contributions to different air pollutant levels, adopting the Response to Extra-Regional Emission Reductions (RERER) metric developed by the HTAP2 community (Galmarini et al., 2017).

## 2 Materials and methods

In the framework of the AQMEII3 project, 12 groups contributed to the simulation of the air pollution levels for 2010 in Europe (EU) and 3 groups for North America (NA) (Table 1 and Solazzo et al., 2017b). As seen in Table 1, different groups used same CTM models, such as the Community Multi-scale Air Quality (CMAQ) model and Weather Research and Forecasting model with chemistry (WRF-Chem) model. The main differences among these models reside in the number of vertical levels, horizontal spacing, biogenic emissions, gas/aerosol modules in the models and the model releases (Table 1). For example, regarding groups that used the CMAQ model, UK1, DE1 and US3 calculated biogenic emissions using the Biogenic Emission Inventory System (BEIS) version 3 model, while TR1, UK1 and UK2 calculated biogenic emissions through the Model of Emissions of Gases and Aerosols from Nature (MEGAN) (Guenther et al., 2012). Moreover, DE1 does not include the dust module, while the other CMAQ instances use the inline calculation (Appel et al., 2013), and TR1 uses the dust calculation previously calculated for AQMEII phase 2. Finally, all runs were carried out using CMAQ version 5.0.2, except for TR1, which is based on the 4.7.1 version. The gas-phase mechanisms and the aerosol models used by each group are also presented in Table 1. IT1 used the WRF-Chem model version 3.6, with a new chemistry that includes a better representation of the secondary organic aerosol mass in the simulation of direct and indirect aerosol effects (Tuccella et al., 2015). In addition, only direct effects were included in the IT1 simulation. The ES1 model also used WRF-Chem, with different gas-phase chemistry. More details of the model system are provided in the Supplement of Im et al. (2018).

The emission inventories that are used in the second phase of AQMEII for Europe and North America (Im et al., 2015a, b) and extensively described in Pouliot et al. (2015) are also used in AQMEII3. For the EU, the 2009 anthropogenic emission inventory from the Monitoring Atmospheric Composition and Climate (MACC) was used. For the NA domain, the 2008 National Emissions Inventory was used with 2010-specific adjustments for major point sources, mobile sources and wildfires (Pouliot et al., 2015). The emissions were then treated with the SMOKE emission processing system (Mason et al., 2012). The majority of the European groups used MACC emissions over Europe, while FI1 and FRES1 supplemented the MACC emissions with HTAP emissions over north Africa (Table 1). For NA, the temporal and vertical allocation of emissions varies between the groups that used the SMOKE files (DE1, US1, US3) and the gridded HTAP files (DK1); however, the annual total mass is exactly the same. In order to guarantee consistency between the groups using the regional-scale MACC or SMOKE emissions, and the groups using the HTAPv2.2 emissions, the regional-scale emission inventories were embedded in the HTAPv2.2 inventory (Janssens-Maenhout et al., 2015; Galmarini et al., 2017). Overall, there was a high level of harmonization of emission inputs even if there were some differences in how they were adapted by each modeling group for their system. Chemical boundary conditions for both domains were provided by the European Centre for Medium-Range Weather Forecasts (ECMWF) Composition - Integrated Forecast System (C-IFS) model (Flemming et al., 2015).

### Emission perturbations

The perturbation scenarios feature a reduction of 20 % of the anthropogenic emissions globally and in HTAP-defined regions of Europe, North America and East Asia (Table 2 and Fig. S1 in the Supplement). The choice of 20% was motivated by the consideration that the perturbation would be large enough to produce a sizeable impact (i.e., more than numerical noise) even at long distances, while small enough to be in the near-linear atmospheric chemistry regime (Galmarini et al., 2017). The emission reductions are implemented in both the global C-IFS model that provides the boundary conditions to the participating regional models, as well as in the regional models. The regional models use the corresponding set of boundary conditions extracted from the C-IFS model. Among the 14 groups that participated in the AQMEII3 base case simulations, 12 groups from Europe and 2 groups from North America simulated at least one of the three emission perturbation scenarios shown in Table 1. Two of the European groups (DE1 and DK1) also simulated the base and the three perturbation scenarios for the North American domain.

– The global perturbation scenario (GLO) reduces the global anthropogenic emissions by 20%. This change has been implemented in the C-IFS global model that provides the boundary conditions to the regional models participating in the AQMEII ensemble. Therefore, the GLO scenario introduces a change in the boundary conditions as well as a 20 % decrease in the anthropogenic emissions used by the regional models. Nine groups over the EU domain and four groups over the NA domain have simulated the GLO scenario.
– The North American perturbation scenario (NAM) reduces the anthropogenic emissions in North America by 20 %. This change has been implemented in the C-IFS global model that provides the boundary conditions to the regional models used in the AQMEII ensemble. Therefore, the NAM scenario introduces a change in the boundary conditions, while anthropogenic emissions remain unchanged for Europe, showing the impact of long-range transport of North American pollutants to Europe, while for North America the scenario introduces a 20 % reduction of anthropogenic emissions in the HTAP-defined North American region, showing the contribution from the domestic anthropogenic emissions. Seven groups over the EU domain and three groups over the NA domain have simulated the NAM scenario.
– The European perturbation scenario (EUR) reduces the anthropogenic emissions in the HTAP-defined European domain by 20%. The EUR scenario introduces a change in the anthropogenic emissions over the EUR region in the CTMs, showing the contribution from the domestic anthropogenic emissions. Six groups have simulated the EUR scenario over the EU domain.
– The East Asian perturbation scenario (EAS) reduces the anthropogenic emissions in East Asia by 20 %. Similar to the NAM scenario for the EU domain, the EAS scenario introduces a change in the boundary conditions, while anthropogenic emissions remain unchanged in the regional models, showing the impact of long-range transport from East Asia on the NA concentrations. Four groups have simulated the EAS scenario over the NA domain.

In AQMEII, all participating groups were required to upload modeled hourly surface concentrations to the ENSEMBLE system at EC-JRC, at specified monitoring stations in EU and NA, as well as surface gridded data (Galmarini et al., 2012; Im et al., 2015a, b; Solazzo et al., 2017b). This study investigates the impacts of emission perturbations and boundary conditions on O_3_, NO_2_, CO, SO_2_, PM_10_ and PM_2.5_ levels over Europe and North America.

Differences between each perturbation scenario and the base case (C-IFS global and regional models run with baseline emissions) are calculated from the gridded hourly pollutant fields, which are then monthly and annually averaged in order to estimate the impact of the perturbation of the corresponding emission or boundary condition.

To estimate the contribution of foreign emission perturbations relative to the GLO perturbation, we have also calculated the RERER metric (Galmarini et al., 2017; Huang et al., 2017; Jonson et al., 2018). For Europe, RERER is calculated using the differences between the GLO vs. BASE as well as the differences between EUR vs. BASE simulations for Europe (Eq. 1), while for North America RERER is calculated using the differences between the GLO vs. BASE and NAM vs. BASE simulations (Eq. 2):

(1) $${\mathrm{RERER}}_{EUR}=\frac{R_{\mathrm{GLO}}-R_{\mathrm{EUR}}}{R_{\mathrm{GLO}}}$$

(2) $${\text{RERER}}_{\mathrm{NAM}}=\frac{R_{\text{GLO}}-R_{\text{NAM}}}{R_{\text{GLO}}},$$

where *R*_GLO_ is the response of the concentration of a given species to global emission reduction, *R*_EUR_ is the response of a concentration of a species to the EUR perturbation for the European domain, and *R*_NAM_ is the response of a concentration of a species to the NAM perturbation for the North American domain. Therefore, a subset of modeling groups that have conducted the three simulations (BASE, GLO and EUR/NAM for Europe and North America, respectively) has been used in the metric calculations (see Table 1). The higher the local response, the smaller the RERER metric. The RERER value can exceed a value of 1 when emission reductions lead to increasing concentrations (e.g., O_3_ titration by nitrogen monoxide, NO).

## 3 Results

### 3.1 Model evaluation

The base case simulation of each model has been evaluated on a monthly mean basis using available surface observations from Europe and North America. The observational data used in this study are the same as the dataset used in the second phase of AQMEII (Im et al., 2015a, b). The data were provided from the surface air quality monitoring stations operating in EU and NA. In EU, surface data were provided by the European Monitoring and Evaluation Programme (EMEP, 2003; http://www.emep.int/, last access: 25 June 2018) and the European Air Quality Database (AirBase; http://acm.eionet.europa.eu/databases/airbase/, last access: 25 June 2018). NA observational data were obtained from the NAtChem (Canadian National Atmospheric Chemistry) database and from the Analysis Facility operated by Environment Canada (http://www.ec.gc.ca/natchem/, last access: 25 June 2018).

**Figure 1.**
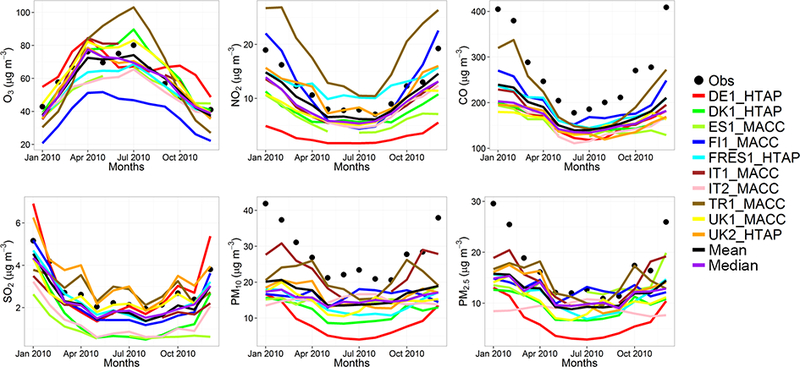
Observed and simulated monthly mean air pollutant levels, averaged over the monitoring stations over Europe.

**Figure 2.**
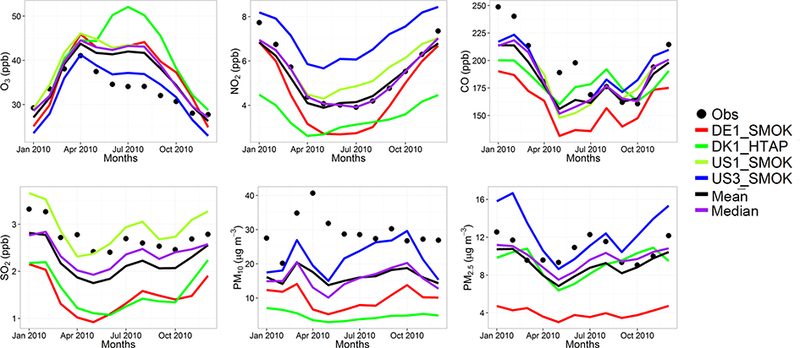
Observed and simulated monthly mean air pollutant levels, averaged over the monitoring stations over North America.

The model evaluation results for each model are presented in Figs. 1 and 2, and in Table 3, along with the results for the multi-model (MM) mean and median values. The results show that the monthly variations of gaseous pollutants are well captured by all models with correlation coefficients (r) generally higher than 0.70. The biases in simulated O_3_ levels are generally less than 10 % with a few exceptions of up to −35 %. The temporal variations of NO_2_ levels are also well simulated (*r* > 0.7) but exhibit much higher biases, with underestimations up to 75 %. CO levels are underestimated by up to 45 %, while a majority of the models underestimated SO_2_ levels by up to68%. Few models overestimated SO_2_ by up to 49 %. PM_10_ and PM_2.5_ levels are underestimated by 20 to 70 %. Slightly higher biases are calculated for the PM_10_ levels.

The model biases can be attributed to meteorology, in particular wind speed and planetary boundary layer (PBL) height, as well as the aerosol mechanisms used in different models that can underestimate either the inorganic aerosols (e.g., IT2) or the secondary organic aerosols (e.g., DK1), leading to underestimations in simulated PM mass. As discussed in Solazzo et al. (2017b), the EU3 region that covers the central Europe including the Alps has the largest errors in terms of wind speed, mainly attributed to the diurnal component of the error, with some models having also large errors in the synoptic component. This region also represents the lowest correlation coefficients for all models. They further conclude that emissions and their vertical distribution are the main source of model biases, in particular for the primary species such as CO and PM. Regarding O_3_, they found that the models have highest biases in the large-scale synoptic component, while the diurnal variations are well captured in general. A more comprehensive evaluation of the models is presented in Solazzo et al. (2017b), Galmarini et al. (2017) and Im et al. (2018).

C-IFS base case results have also been evaluated along with the regional CTMs, as presented in Figs. 1 and 2, and in Table 3. The seasonal variations for O_3_, NO_2_, CO and SO_2_ are well captured with high correlation values of ~ 0.9. PM_10_ and PM_2.5_ showed a different seasonal cycle than the observation by not reproducing the wintertime maxima (*r* = ~ −0.7). The C-IFS model underestimates O_3_ and CO by ~ 20 % over Europe, while NO_2_ is slightly overestimated (NMB of 7 %). SO_2_ is overestimated by ~ 10 % over Europe, while PM_10_ and PM_2.5_ levels are largely underestimated by ~ 60%, which can be attributed to the lack of secondary aerosol mechanism in the bulk C-IFS model. Over the North American domain, C-IFS well captures the seasonal variations of O_3_, NO_2_ and CO with correlation coefficients larger than 0.7, while the seasonal variation of SO_2_ is not captured by the model (*r* = 0.04). The seasonal variations of PM_10_ and PM_2.5_ are also poorly captured (*r* < 0.2). North American O_3_ levels are slightly underestimated (NMB of −10 %), while NO_2_ and CO are overestimated by ~ 40 and 20 %, respectively. SO_2_ is overestimated by 35 %, while PM_10_ is largely underestimated by ~ 80 and PM_2.5_ by ~ 40 %. Over both Europe and North America, the wintertime PM levels are underestimated due to lack of secondary aerosols, while the spring-summer peaks are attributed to long-range transport of desert dust from the Sahara, which affects mainly the southeast region of North America.

### 3.2 Perturbation analyses

**Figure 3.**
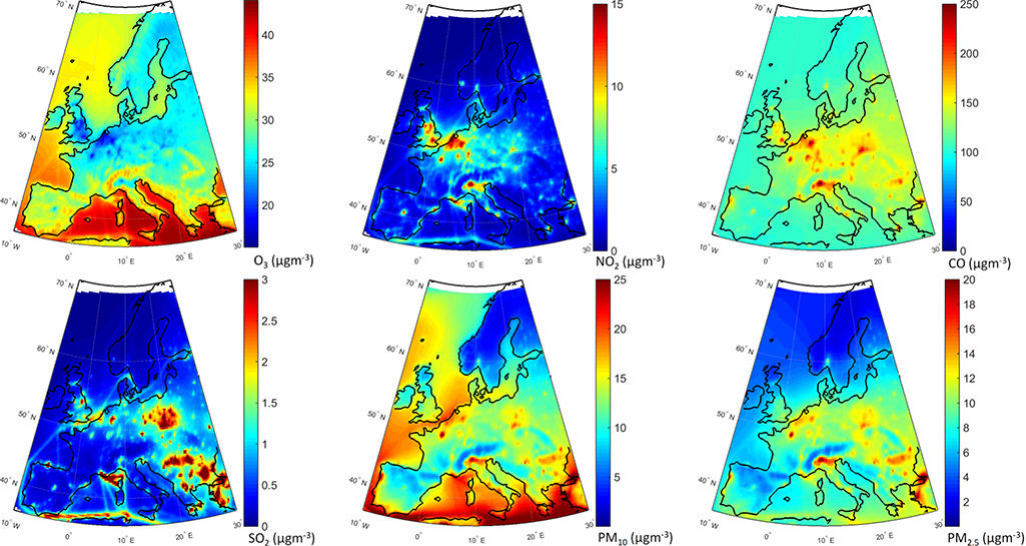
Multi-model mean air pollutant levels over Europe as simulated in the base case.

**Figure 4.**
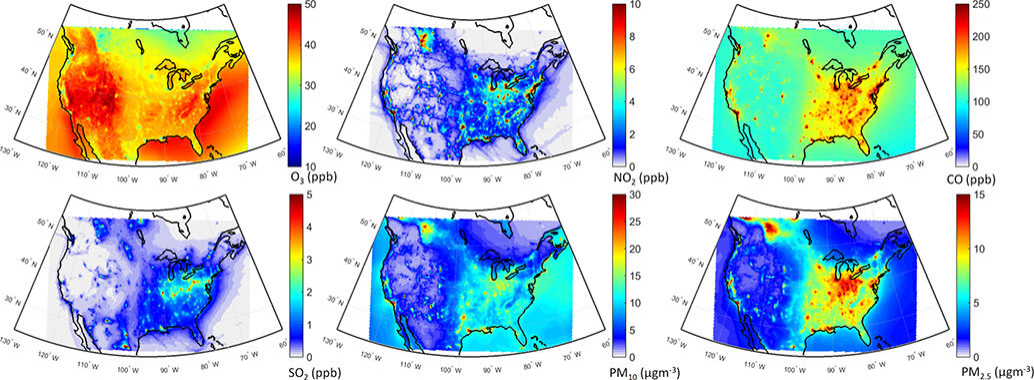
Multi-model mean air pollutant levels over North America as simulated in the base case.

The annual mean relative differences of each perturbation scenario from the base case scenario, averaged over all stations, are provided in Table 4 (EU) and Table 5 (NA) for each modeling group, along with the results for the MM ensemble mean and median. The base case monthly mean time series for the participating groups are provided in Figs. 1 and 2 for each pollutant, while Figs. 3 and 4 shows the annual mean spatial distribution of the pollutants from the MM ensemble mean calculations over Europe and North America, respectively. As seen in the time series figures, there is a large spread among different groups due to the different models used and the different sets of anthropogenic emissions (Table 1). However, the temporal variation is consistent among all models, in particular for the gaseous species.

#### 3.2.1 Impact of the global emission reduction scenario (GLO)

##### Europe

**Figure 5.**
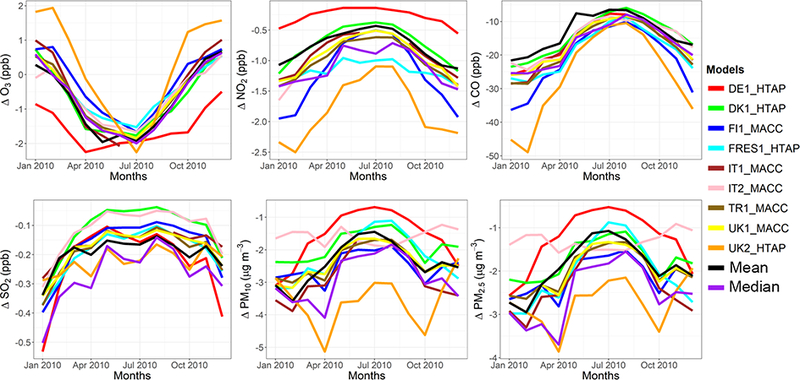
Absolute impact of the 20 % reduction of the global anthropogenic emissions over Europe (GLO_EUR_ – BASE_EUR_).

The monthly time series of the differences between the GLO and the BASE simulations for each pollutant are presented in Fig. 5. The annual differences are reported in Table 4. Regarding the primary gaseous pollutants, all models simulate the smallest differences during the summer months, while the differences are largest in winter. For O_3_, the simulated differences are positive in winter and negative in summer for all models except for DE1 that simulated a decrease in all months. Results suggest that wintertime O_3_ over Europe is mainly controlled by anthropogenic emissions. For the other pollutants, results suggest that their levels are mainly controlled by anthropogenic emission throughout the year. The annual difference is smallest for O_3_, with a reduction of −0.34 ± 1.23ppb (−1.04 ± 4.00%). The annualmean value of the O_3_ daily maximum of8h running average decreases by −0.53± 1.50ppb (−1.62±3.99%). NO_2_ levels decreased by 0.97 ± 0.45ppb (19.34 ± 1.59%) over Europe, while CO levels decreased by 17.35 ± 4.03ppb (11.22 ± 1.17%), SO_2_ levels by 0.18 ± 0.05ppb (20.87 ± 0.93%), PM_10_ by 2.38 ± 0.68μg m^−3^ (15.84 ± 2.12%) and PM_2.5_ by 2.02 ± 0.52 μg m^−3^ (18.30 ± 1.75%). Vivanco et al. (2018) found similar reductions regarding the deposition of sulfur and nitrogen species over Europe. Almost all models simulate an overall decrease of annual mean O_3_ levels over EU (−0.94 to −4.65%), with the exception of TR1 that simulated an increase of 9.31%. Regarding other pollutants, all models simulate a decrease during the simulation period. In general, DE1 and TR1 model groups stand out for introducing the smallest and largest differences, particularly for O_3_, NO_2_ and PM.

**Figure 6.**
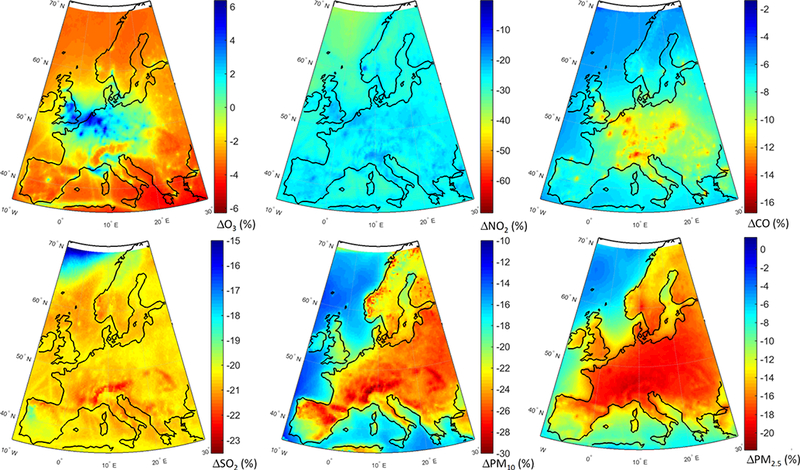
Spatial distribution of the annual mean relative differences between the global perturbation scenario and the base case over Europe as simulated by the multi-model mean ensemble.

The geographical distribution of the change in annual mean concentrations in the GLO scenario as simulated by the MM mean is presented in Fig. 6. Regarding O_3_, most of Europe is characterized by decreased concentrations (Fig. 6a). Over central Europe, where most of the primary emissions are located (e.g., NO_X_), O_3_ levels slightly increase by ~ 2%. Emission hot spots, in particular the Benelux area, stand out with largest increases (~ 6 %) due to a decreased NO_X_ titration effect, which can also be seen in Fig. 6b. In addition, O_3_ levels over the northern parts of Germany and France, and southern UK are increasing in response to emission reductions. There is also a clear decrease in CO levels (Fig. 6c), in particular over central Europe by up to ~ 16 %. All primary species decrease over the whole domain, especially over the industrial hot spots such as in Poland, the Po Valley and the Benelux area (Fig. 6d). PM levels decrease throughout the domain by up to ~ 20 % (Fig. 6e and f).

##### North America

**Figure 7.**
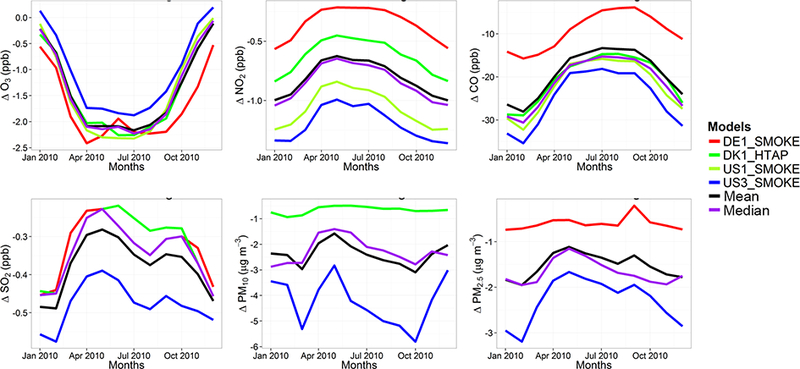
Absolute impact of the 20 % reduction of the global anthropogenic emissions over North America (GLO_NAM_ – BASE_NAM_).

The seasonal variations of the impact of 20 %-decreased global emissions on the North American pollutant levels are presented in Fig. 7. All models simulated a small decrease of 3 to 5 % (Table 5) in O_3_ levels with the largest differences in spring to summer (Fig. 7a). The mean response to the emission perturbation is estimated to be-1.39 ± 0.27 ppb (−3.52 ± 0.80%). The annual mean value of the O_3_ daily maximum of 8 h running average decreases by −1.93 ± 0.14 ppb (−4.51 ± 0.45%). All models simulated the largest NO_2_ response in winter. Most models simulated a decrease of NO_2_ levels, while DK1 estimated an increase (Fig. 7b). As shown in Table 5, the models simulated a NO_2_ response of ~ 0.4–1.2ppb (−17.8 ± 0.78 %). Regarding CO, all models simulated a very clear seasonal profile of the response to emission reductions, with maximum change in late winter/early spring and the minimum change in summer. Most models simulated a change around −15 to −25 ppb (~ 11 %), with the exception of the DE1 model simulating a decrease of ~ 9 ppb (~ 7.9 %). The MM mean response is calculated to be 19.2 ± 6.9 ppb (−11 ± 2.3%). The impact of the emission reduction on SO_2_ levels was calculated to be −0.25 to −0.48 ppb (−20.3 ± 0.2 %).

The response of PM_10_ levels to the global emission reduction was calculated to be −2.4 ± 1μgm^−3^ (−32.1 ± 26.6 %) (Table 5). The largest relative change was calculated for DE1 (~ 63%). DK1 has almost a flat response around −1 μgm^−3^, while DE1, which is overlapped with the median line, and US3 have maximum responses in early spring and mid-autumn, while they simulate a minimum response in winter and late spring. Regarding PM_2.5_, the multi-model mean response was calculated to be −1.5 ±0.9μgm^−3^ (−17.2± 1.8 %). DK1 (overlapped with the median) and US3 simulated the minimum response in May (Fig. 7f), while US3 has a slightly higher second minimum in September. This minimum is also simulated by DE1 as the minimum response. DE1 simulates the lowest response among the three models.

**Figure 8.**
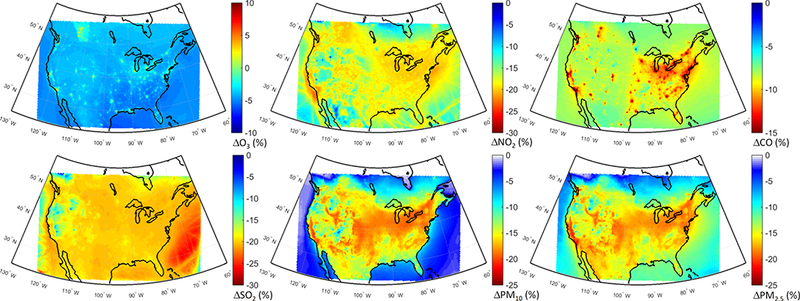
Spatial distribution of the annual mean relative differences between the global perturbation scenario and the base case over North America as simulated by the multi-model mean ensemble.

The spatial distributions of response of different pollutants to the GLO scenario are presented in Fig. 8. O_3_ levels are reduced over most of the domain (Fig. 8a), with slight increases over the emission hot spots due to a reduced effect of NO_x_ titration, as seen in Fig. 8b, as well as decreased CO levels over the whole domain (Fig. 8c). SO_2_ levels are also decreased throughout the domain (Fig. 8d), with the largest reductions over the Atlantic (attributable to reduction in shipping emissions). The western part of the continent is characterized by the lowest reductions. PM levels are reduced throughout the domain by up to 25 % (Fig. 8e and f), with the largest reductions over the eastern and central parts of the domain. A large decrease, more pronounced in the PM_2.5_ response, can also be seen over California in the western coastal United States.

#### 3.2.2 Impact of the North American emission reduction scenario (NAM)

##### Europe

NA emission reductions account for a reduction of European O_3_ levels of −0. 22 ± 0.07ppb (−0.75 ± 0.14%), with all models simulating a decrease of −0.51 to 0.86%, except for the ESI model that simulated an increase of 1.31% (Table 4). This decrease is in agreement with previous studies, such as the HTAP2 study (UN, 2017) that calculated an O_3_ reduction over Europe of 0.22ppb in response to a 20% decrease in the North American NOx emissions, and Fiore et al. (2009) that simulated a MM mean response of −0.4ppb in response to a 20% reduction of anthropogenic emissions in North America. NO_2_ levels increase slightly by 0.16 ± 0.01%. The annual mean value of the O_3_ daily maximum of 8h running average decreases by −0.15 ± 0.27ppb (−0.45 ± 0.77%). CO levels also decreased over the EU domain by −1.39 ± 0.27ppb (−0.96 ± 0.22%), much higher than ~ 0.1ppb calculated by Fiore et al. (2009). PM_10_ and PM_2.5_ levels also decreased slightly by −0.03 ± 0.03μgm^−3^ (−0.21 ± 0.7%) and −0.02 ± 0.02μgm^−3^ (−0.18 ± 0.25%), respectively. The models had different SO_2_ responses to the NA emissions. Overall, DE1, ES1 and FRES1 simulated almost no change in the surface SO_2_ levels, while DK1, ES1 and TR1 simulated an increase (0.10, 5.75 and 0.01 %, respectively) and FI1 and UK1 simulated a decrease (−0.02 and −0.03 %, respectively). Different responses can be due to different model setups including aqueous chemistry, vertical resolutions and aerosol modules (Solazzo et al., 2017b).

**Figure 9.**
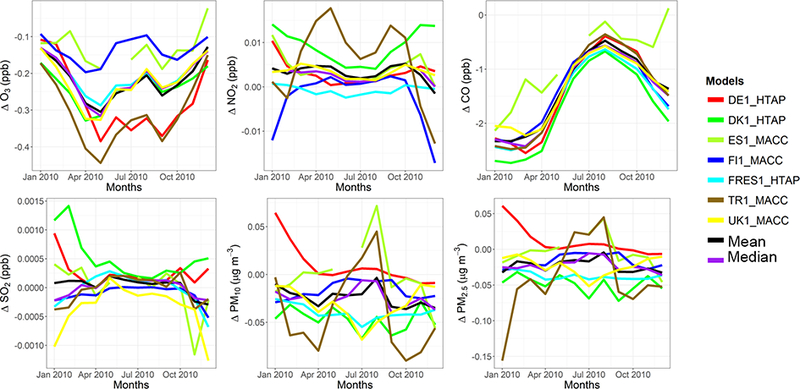
Absolute impact of the 20 % reduction of the North American anthropogenic emissions over Europe (NAM_EUR_ – BASE_EUR_).

All models were consistent in simulating the largest impact on O_3_ during spring and a second lower peak in autumn (Fig. 9a). Surface mean NO_2_ concentrations (Fig. 9b) increased in most models except for FRES1 that simulated a small decrease except for winter. FI1 also simulated a decrease during the winter period extending to the transition periods. All models, except for ES1, simulated a similar response of CO concentrations to perturbation to NA emissions, with a distinct seasonality (Fig. 9c). The SO_2_ response in models is also consistent except for the winter period where there is a large spread in magnitude and the sign of the response (Fig. 9d).

**Figure 10.**
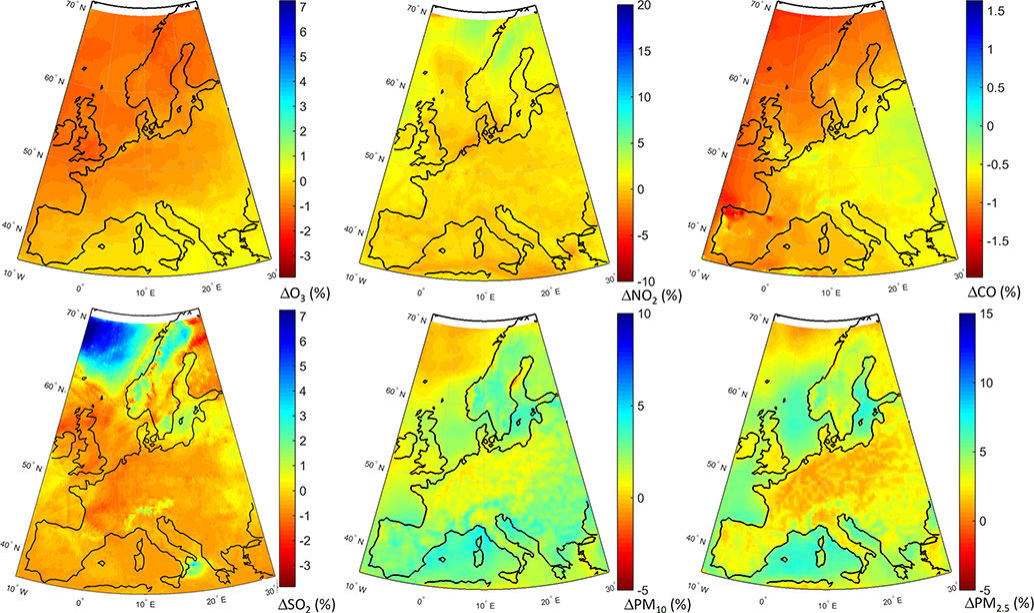
Spatial distribution of the annual mean relative differences between the North American emission perturbation scenario and the base case over Europe as simulated by the multi-model mean ensemble.

O_3_ levels decreased slightly over the entire European domain by up to 3% (Fig. 10a). The largest impact is simulated over the western boundary and gradually decreases eastwards. The response of O_3_ levels to NAM emissions is more evident during spring where there is a clear transport from the Atlantic to the western/northwestern parts of Europe such as the UK, northern France and Scandinavia (Fig. S2a). The transport of Atlantic air masses is also shown for the springtime CO levels over Europe (Fig. S2b). The ensemble mean simulates a slight increase of up to 3% in NO_2_ levels over Europe (Fig. 10b). Along with the O_3_ levels, CO levels show the largest decrease over northwestern Europe by up to ~ 2%. SO_2_ levels increased over the whole domain, in particular over eastern Europe and the Alpine region (Fig. 10d), due to a decrease in the oxidative capacity of the atmosphere (see Fig. 10a for O_3_), leading to a decrease in the SO_2_ to SO4 conversion. This results in an increase of the SO_2_ levels and a decrease in the PM_2.5_ levels (Fig. 10e and f).

##### North America

The response of North American pollutant levels to a 20 % reduction of North American anthropogenic emissions (implemented in both C-IFS and the regional CTMs) is presented in Table 5. The NAM scenario led to a decrease of annual mean O_3_ levels over North America by −0.36ppb (US3) to −0.92ppb (DE1), with MM ensemble mean calculated to be −0.65 ± 0.28ppb (−1.45 ± 0.88%), in agreement with Fiore et al. (2009) that calculated a decrease of ~ 1 ppb. The annual mean value of the O_3_ daily maximum of 8h running average decreases by −1.11 ± 0.11 ppb (−2.60 ± 0.36%), very similar to the change over Europe. Consequently, the largest change in NO_2_ levels was simulated by US3 (−1.17ppb) and smallest by DE1 (−0.36ppb). The MM mean response of NO_2_ is calculated to be −0.71 ± 0.41 ppb (−17.24 ± 0.58 %). Similar to NO2, the largest response in CO levels was simulated by US3 (−19.87ppb) and the smallest by DE1 (−3.84ppb), leading to a MM mean response of −12.35 ± 8.06ppb (−7.01 ± 3.60%). As seen in Table 5, DE1 simulated a much lower absolute and relative change in CO response compared to DK1 and US3. SO_2_ levels decreased by −0.12 to −0.18 ppb, leading to a MM mean response of −0.14 ± 0.08 ppb (−4.23 ± 0.18%). PM_10_ levels decreased −1.78 ± 2.08 μgm^−3^ (−15.78 ± 3.26 %). As seen in Table 5, DK1 simulated a very low response to the NAM scenario, by ~ 0.60μgm^−3^, compared to the DE1 and the US3 groups that simulated a PM_10_ response of −2.02 and −4.19 μgm^−3^, respectively. However, the relative responses are not very different between the different groups (~ 16 %).

**Figure 11.**
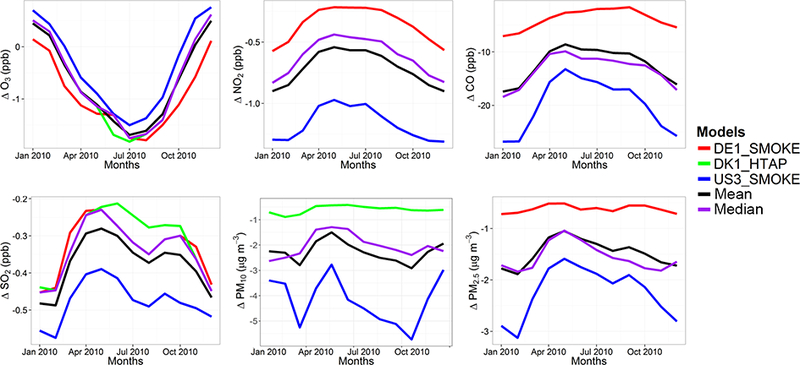
Absolute impact of the 20% reduction of the North American anthropogenic emissions over North America (GLO_NAM_ - BASE_NAM_^)^.

The response of O_3_ to the NAM scenario is largest in summer (Fig. 11a): June for DK1 and US3 and August for DE1. The O_3_ response clearly shows a difference from the GLO response in spring, suggesting the impact of long-range transport in spring that does not appear in the perturbation of the local emissions only. The largest NO_2_ response (Fig. 11b) is simulated by US3, similar to the response to the GLO scenario. The response of CO to the reductions in local emissions (Fig. 11c) is different from the response to the global reduction, where DK1 and US3 have the minimum response in spring and DE1 has the minimum response in autumn. The responses of SO_2_ and PM to GLO and NAM are similar, suggesting the main drivers of SO_2_ and PM levels are local emissions.

**Figure 12.**
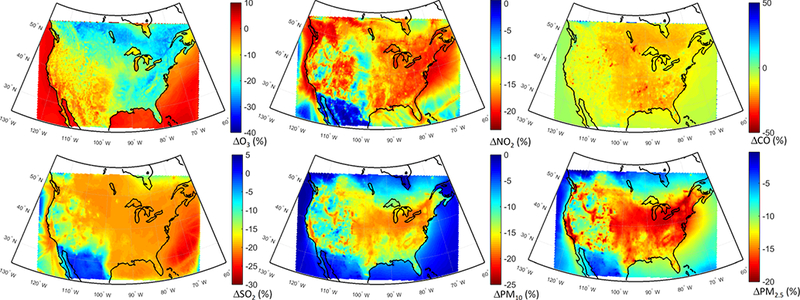
Spatial distribution of the annual mean relative differences between the North American emission perturbation scenario and the base case over North America as simulated by the multi-model mean ensemble.

Annual mean O_3_ levels show large reductions (~ 20 %) over the eastern parts of the domain, while there are slight increases or less pronounced decreases over the western parts of the domain (Fig. 12a) associated with larger NO_x_ reductions (Fig. 12b). CO and SO_2_ levels are mostly reduced over the central to eastern parts of the domain (Fig. 12c and d, respectively), with shipping impacts over the Atlantic being more pronounced on SO_2_ levels. The western parts of the US experience smaller SO_2_ reductions (~ 5–10 %) and slight increases over the southwestern US. The response of PM to the NAM scenario (Fig. 12e and f) is very similar to the response to the GLO scenario (Fig. 8e and f).

#### 3.2.3 Impact of the European emission reduction scenario (EUR)

**Figure 13.**
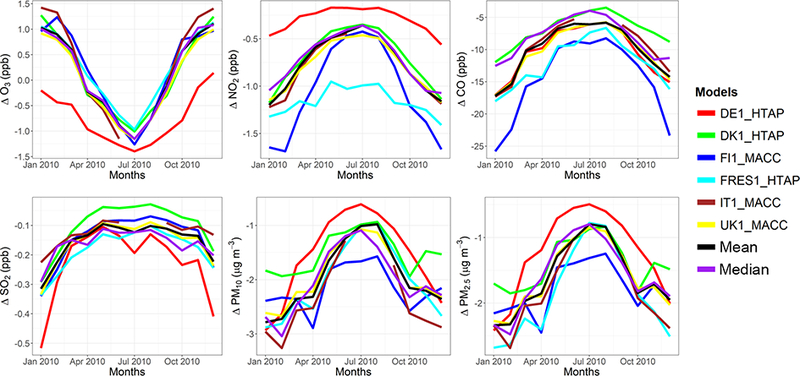
Absolute impact of the 20 % reduction of the European anthropogenic emissions over Europe (EUR_EUR_ – BASE_EUR_).

O_3_ levels increase slightly by 0.01 ± 0.40 ppb (0.25 ± 1.35%) in response to the 20% reduction of the anthropogenic emissions from Europe (Table 4). This response is much lower than Fiore et al. (2009) that calculated a MM mean response of 0.8ppb. However, as seen in Fig. 13a, the positive mean response together with the large standard deviation is due to the DE1 model that simulated a decrease (−2.33 %), while other groups simulated an increase (0.39 to 1.72%). There is a distinct seasonality in the response with winter levels increasing with reduced emissions and summer levels decreasing, following the emission temporal variability. The annual mean value of the O_3_ daily maximum of 8 h running average decreases by −0.21 ± 0.10ppb (−0.62 0.24%). NO_2_ concentrations decreased by −0.75 ± 0.26 (17.68 ± 0.90 %), with a similar seasonal response of SO_2_ levels (−17.52 ± 1.70%) and CO levels (−6.26 ± 1.07%), consistent with the findings of Vivanco et al. (2018). An opposite seasonal variation is calculated for the O_3_ response (Fig. 13b-d), The DE1 model also stands out in the NO_2_ response together with the FRES1 model in the magnitude of the response (Fig. 13b). PM_10_ and PM_2.5_ levels have similar responses to the emission reduction (−14.43 ± 2.84 and −15.67 ± 2.12 %, respectively) with similar seasonality.

**Figure 14.**
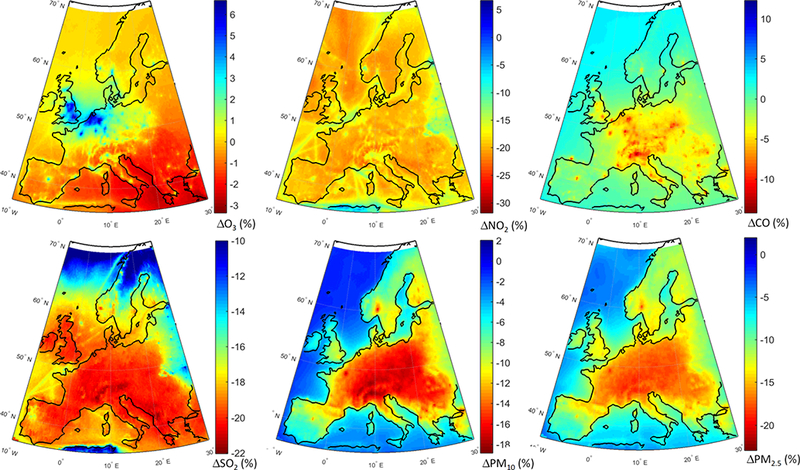
Spatial distribution of the annual mean relative differences between the European emission perturbation scenario and the base case over Europe as simulated by the multi-model mean ensemble.

The MM mean geographical distribution of the O_3_ response is very similar to that of the GLO perturbation (Fig. 14a), with relatively smaller decreases by up to ~ 3%. O_3_ levels increase over central and in particular over northwestern Europe by up to ~ 6%. NO_2_ levels decrease uniformly over the entire domain by up to ~ 20% (Fig. 14b). CO levels decrease over the emission sources, mainly over central and eastern Europe (Fig. 14c). PM levels also decrease over the entire domain, especially over central and eastern Europe (Fig. 14e and f).

#### 3.2.4 Impact of the East Asian emission reduction scenario (EAS)

As seen in Table 5, the impacts of East Asian emissions on North American O_3_ levels are much lower than the impacts from the reductions in global and local emissions. The largest impact is simulated by DE1 as −0.99ppb (−0.35 %), while other models give similar responses (~ 0.60ppb; −0.20%). The O_3_ response as calculated by the MM mean ensemble is −0.25 ± 0.07ppb, in agreement with the HTAP2 findings and Fiore et al. (2009). The annual mean value of the O_3_ daily maximum of 8h running average decreases by −0.28 ± 0.07ppb (−0.65 ± 0.20%). NO_2_ and SO_2_ responses to reductions in EAS emissions were simulated to be very small (−0.04 ± 0.08 and 0.01 ± 0.02%, respectively). The CO response to EAS was simulated to be −2.60ppb (DE1) to −4.16ppb (DK1), with the MM mean response of −3.37 ± 0.68ppb (−2 ± 0.29%). Regarding PM10, DE1 simulated a very large response (~ − 0.56μgm^−3^) compared to DK1 and US3 (~ − 0.05μgm^−3^), leading to a MM mean response of −0.21 ±0.30μgm^−3^ (−5.63 ± 8.50%). However, the PM_2.5_ response was much lower (−0.02±0.03μgm^−3^; −0.20 ± 0.35 %), suggesting that the PM_2.5_ levels are largely driven by local emissions.

**Figure 15.**
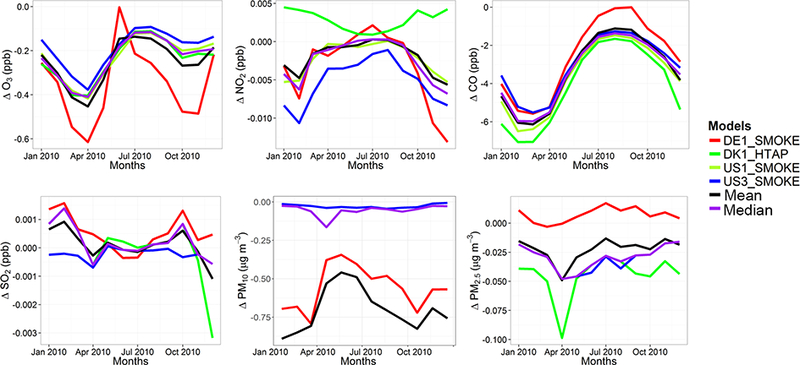
Absolute impact of the 20 % reduction of the East Asian anthropogenic emissions over North America (GLO_NAM_ – BASE_NAM_).

The O_3_ response to EAS emission reductions was highest in spring and autumn, suggesting that long-range transport is important in these seasons (Fig. 15a). The NO_2_ response was negative, being maximum in winter and minimum in summer, except for DK1 showing an increase in NO_2_ levels in all seasons (Fig. 15b). The impact of EAS emissions on North American CO levels showed a distinct seasonality (Fig. 15c), similar to the impact of the global emission reductions (Fig. 5c), suggesting that regional CO levels over North America are driven by both local emissions and long-range transport. The response of SO_2_ to East Asian emission reductions varied largely from model to model, with US3 showing an overall reduction, while DE1 and DK1 simulated increases in winter, spring and autumn, and decreases in summer (Fig. 15d). The PM_10_ responses simulated by DK1 (overlapped with the median) and US3 were simulated to be small, being largest in spring (Fig. 15e). However, DE1 simulated a large and opposite response, with spring having the smallest response and winter with the largest response. DE1 also simulated a different PM_2.5_ response in terms of the sign of the change and thus seasonality in response to DK1 and US3 (Fig. 15f). Largest differences were simulated in spring, similar to PM_10_ by DK1 and US3, while DE1 simulated the largest response in winter and summer, and the spring response was minimum.

**Figure 16.**
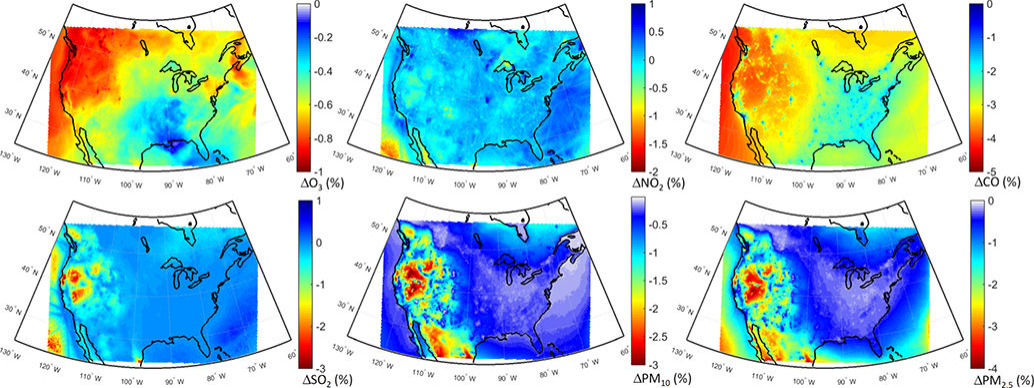
Spatial distribution of the annual mean relative differences between the East Asian emission perturbation scenario and the base case over North America as simulated by the multi-model mean ensemble.

The impact of the East Asian emissions over the western parts of North America is clearly seen for all pollutants in Fig. 16. The impacts are low for all pollutants, being up to 5 %. The impacts are particularly pronounced for CO (Fig. 16c), SO_2_ (Fig. 16d) and PM (Fig. 16e and f). The largest O_3_ response was simulated over the northwestern parts of North America (Fig. 16a). The springtime transport of O_3_ from East Asia is more evident compared to the annual average of the perturbation response (Fig. S3a), where the western NA O_3_ levels decrease by up to ~ 1.5 %. The springtime CO levels also decrease by up to 6 % (Fig. S3b), showing the importance of long-range transport from East Asia.

#### 3.2.5 RERER analyses

As discussed in Sect. 2, the RERER metric (Galmarini et al., 2017; Huang et al., 2017; Jonson et al., 2018) is designed to quantify the relative impact of local vs. non-local emission sources on pollutant levels in the receptor regions (EU and NA). The RERER metrics for EU have been calculated using gridded annual mean pollutant concentrations from the BASE, GLO and EUR simulations for the individual groups as well as for the ensemble mean. For the NA domain, the RERER metrics have been calculated using the annual mean concentrations from the BASE, GLO and NAM simulations. Table 6 presents the RERER metric calculated for the European domain. The table shows differences in the strengths of non-local source contributions to different species among the different models. Regarding the RERER metric for O_3_ in Europe, most values calculated are below 1, except for the IT1 model, which shows a significant increase of O_3_ levels in Europe in response to emission reductions compared with the other models. A RERER value of 0.8–0.9 is calculated for the majority of models, implying the dominance of non-local sources in Europe, except for the DE1 model, where the RERER value is lower (~ 0.5), giving an equal contribution of local vs. non-local sources in Europe. The MM mean RERER value for O_3_ is ~ 0.8, showing a much larger contribution of non-local sources compared to local sources in Europe. This result is in agreement with, however slightly smaller than, Jonson et al. (2018), who calculated a MM mean RERER value of 0.89.

Regarding NO_2_, the RERER metrics (< 0.4) show that NO_2_ is controlled by local sources. In addition, the RERER metrics calculated for DE1 and FI1 are slightly negative, implying that the signal is not sensitive to non-local emissions. RERER calculated for the ensemble mean for NO_2_ (~ 0.2) also shows the high sensitivity of NO_2_ concentrations to local sources. The RERER metric calculations for CO shows similar contributions from local vs. non-local sources, with RERER values of 0.4–0.6, except for IT1. IT1 has a RERER metric value of ~ 0.9, suggesting a large contribution of non-local sources, leading to the higher sensitivity of CO to non-local sources compared to other model groups. The RERER values calculated for the ensemble mean (~ 0.6) show a slightly larger contribution of non-local sources compared to local sources. The MM mean RERER value of 0.55 for CO from this study is in very good agreement with Jonson et al. (2018) that calculated a MM mean RERER of 0.51. RERER metrics calculated for SO_2_ are also in the low range (0–0.4). While DE1 and FI1 show almost no signal for the non-local contribution, DK1, IT1 and UK1 are in the higher end of the range. The CO MM mean RERER value of ~ 0.3 shows that CO levels are largely controlled by local emissions. Finally, the metrics calculated for PM_10_ and PM_2.5_ show that local sources are the main contributor to the PM levels in Europe (RERER of ~ 0–0.3), leading to an ensemble mean contribution of local sources (RERER of ~ 0.2).

Regarding the local vs. non-local contributions to different pollutants over the North American domain, three groups out of four simulated the GLO and NAM scenarios needed to calculate the RERER metrics. RERER metrics show that O_3_ is largely controlled by non-local sources. European model groups DE1 and DK1 simulate a larger influence of nonlocal sources (~ 0.8-~ 0.9) compared to the US3 group, which simulated lower RERER metric values of ~ 0.5, indicating that O_3_ levels are driven equally by local and non-local sources. This lower value is also consistent with the findings of Huang et al. (2017), who simulated the largest impacts on O_3_ in May and June with RERER values around ~ 0.5. The ensemble mean shows that O_3_ responses are largely attributable to non-local sources (RERER of ~ 0.8), which are similar to those found for Europe. RERER metric values calculated for NO_2_ by different models (RERER of ~ 0–0.2) and the ensemble mean (RERER of 0.05) clearly show that NO_2_ is controlled by local sources, similar to the European case. The sensitivities of CO to local and non-local sources are similar to those for O_3_, with DE1 and DK1 simulating a large contribution from non-local sources, while US1 shows that CO is controlled equally by local and non-local sources (RERER of 0.5). Similar to NO_2_, all models show that SO_2_ is largely driven by local sources with RERER values between ~ 0.1 and ~ 0.2. Regarding the particles, models simulate very similar responses to changes in the local and nonlocal sources. RERER values are calculated to be ~ 0.08 and ~ 0.11 for PM_10_ and PM_2.5_, respectively, showing the large local contribution compared to non-local sources.

**Figure 17.**
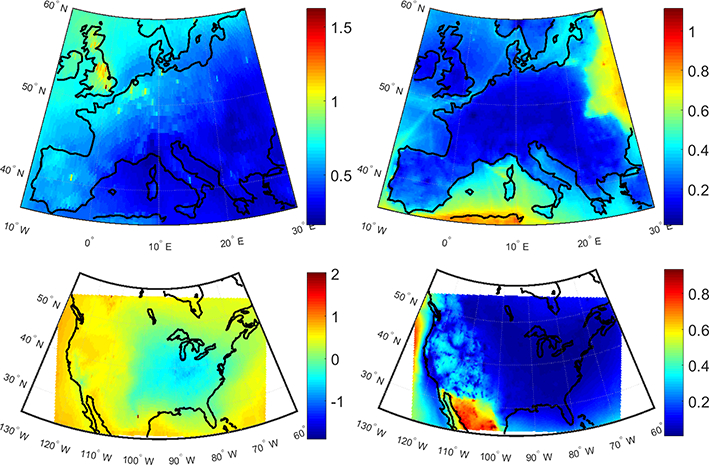
Spatial distribution of RERER values constructed from the annual mean responses of O_3_ and PM_2.5_ over Europe and North America.

Figure 17 shows the spatial distributions of the MMM RERER values for O_3_ and PM_2.5_, as constructed from the annual mean responses to perturbation scenarios over Europe and North America. Figure 17a shows that O_3_ is dominantly controlled by non-local sources with RERER values higher than 0.5 throughout the domain. Higher values are calculated over northwestern Europe, in particular over UK and the northwestern part of the domain covering the Atlantic. In contrary, PM_2.5_ levels are controlled by local sources with RERER values around 0.2 (Fig. 17b). North American O_3_ levels are largely controlled by non-local sources over the western part of the domain, with RERER values above 0.5 (Fig. 17c). Local sources play a more important role in controlling O_3_ levels over the eastern part of the US where much lower RERER values are calculated. PM_2.5_ levels are dominantly controlled by the local sources, similar to the case in Europe, with low RERER values throughout the domain (Fig. 17d). PM_2.5_ levels over the western part of the domain have however a relatively larger contribution from non-local sources. It is important to note that the sharp gradients in the PM_2.5_ RERER values over both the eastern part of the European domain and the Mexican part of the NA domain are due to HTAP2 definition of source regions where the perturbations are introduced. Therefore, due to the large contribution of the local sources to PM_2.5_ levels, large gradients are calculated across the HTAP2 borders. As O_3_ is largely controlled by non-local sources, these gradients do not exist.

**Figure 18.**
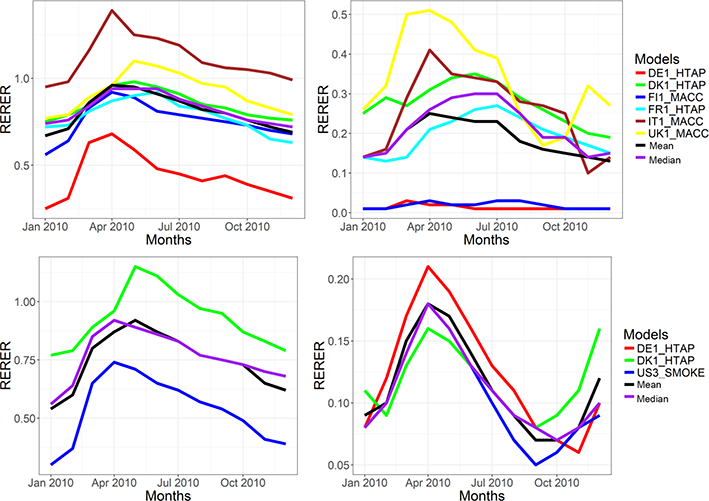
Seasonal variations of RERER values of O_3_ and PM_2.5_ over Europe and North America.

In order to further analyze the impact of local vs. nonlocal sources, the monthly variations of RERER values for O_3_ and PM_2.5_ over both domains are presented in Fig. 18. All models simulate a larger non-local source contribution during the spring period for both domains and pollutants. For both pollutants and domains, the local sources have relatively larger contribution in winter periods, reflected by the lower RERER values compared to other parts of the year. Regarding European O_3_, the majority of the models show a RERER value of between 0.5 and 1, while DE1 shows much lower and IT1 much higher values (see also Table 6). DE1 and FI1 simulate the lowest RERER values for PM_2.5_ (< 0.1), while other models calculate RERER values between 0.1 and 0.5. Regarding O_3_ over North America, US3 shows that, in winter months, O_3_ is controlled more by local emissions with RERER values much lower than 0.5, while DE1 shows the highest non-local contributions throughout the year.

## 4 Conclusions

In the framework of the third phase of the Air Quality Model Evaluation International Initiative (AQMEII3), the impacts of local vs. foreign emissions over the European and North American receptor regions are simulated by introducing a 20 % decrease of global and regional emissions by research groups, using different state-of-the-art chemistry and transport models. The emission perturbations were introduced globally, as well as over the HTAP2-defined regions of Europe, North America and East Asia. The base case and perturbation scenarios are first simulated using the global C-IFS model, which provides the boundary conditions to the regional CTMs.

The base case simulation of each model has been evaluated against surface observations from Europe and North America. The temporal variabilities of all pollutants are well captured by all models with correlations generally higher than 0.70. O_3_ levels are generally simulated with a NMB less than 10 % with few exceptions of NMB values up to −35 %. NO_2_, CO and SO_2_ levels are simulated with underestimations up to 75, 45 and 68 %, respectively. PM_10_ and PM_2.5_ levels are underestimated by 20 to 70 %, with slightly higher biases in PM_10_ levels.

Results from the perturbation simulations show that the largest impacts over both the European and North American domains are simulated in response to the global emission perturbation (GLO). These responses are similar, however slightly lower, compared to the local emission perturbation scenarios for Europe (EUR) and North America (NAM). In contrast to the GLO scenario, O_3_ levels over Europe slightly increase by 0.13 ppb (0.02 %). The annual mean value of the O_3_ daily maximum of 8 h running average decreases in all scenarios over Europe, being highest in the GLO scenario by ~ 1 % and lowest in the NAM scenario by ~ 0.3 %. Over North America, the annual mean value of the O_3_ daily maximum of 8 h running average decreased by ~ 5 % in the GLO scenario, 3 % in the NAM scenario and 0.7 % in the EAS scenario. The impact of foreign emissions simulated by the NAM scenario for Europe and EAS scenario for North America was found to be lowest, however still noticeable, particularly close to the boundaries. This impact is especially noticeable (up to only a few percent) for the western parts of the North American domain in response to the emission reductions over East Asia. The response is almost linear (~ 20 % decrease) to the change in emissions for NO2, SO_2_ and PM in the global perturbation scenario (GLO), while O_3_ levels decrease slightly (~ 1 %).

Despite these small differences, there are large geographical differences. NO_2_, CO and SO_2_ levels are mainly affected over emission hot spots in the GLO scenario as well as in the EUR scenario for Europe and the NAM scenario for North America. O_3_ levels increase over the hot spot regions, in particular the Benelux region in Europe, by up to ~ 6 % due to the reduced effect of NO_x_ titration. Over the North American domain, the central-to-eastern part and the western coast of the US experience the largest response to the global emission perturbation. For most of the pollutants, there is distinct seasonality in the responses particularly to the global and local emission perturbations. The largest responses are calculated during winter months, where anthropogenic emission are highest, except for O_3_, where largest responses are seen during spring/summer months, suggesting photochemistry still plays an important role in O_3_ levels.

The RERER metrics have been calculated to examine the differences in the strengths of non-local source contributions to different species among the different models. The large RERER values over Europe and North America for O_3_ (~ 0.8) show a larger contribution of non-local sources, while for other gaseous pollutants (NO_2_, CO and SO_2_) and particles (PM_10_ and PM_2.5_), low RERER values (< 0.5) indicate that these pollutants are largely controlled by local sources. Results show that the contribution of local sources to NO_2_, SO_2_ and PM levels are larger in North America compared to Europe, while for CO, local sources have a larger share in Europe in comparison with North America. In addition, RERER analyses show that European O_3_ is largely controlled by nonlocal sources (RERER > 0.5) throughout the domain. PM_2.5_ levels are largely controlled by local sources with RERER values around 0.2 throughout the domain. Local sources play a more important role in controlling O_3_ levels over the eastern part of the US. PM_2.5_ levels over the western part of NA have a relatively larger contribution from non-local sources compared to the rest of the domain. A larger non-local source contribution during the spring period for both domains and pollutants has been calculated, suggesting long-range transport from non-local sources. For both pollutants and domains, the local sources have relatively larger contribution in winter periods, reflected by the lower RERER values compared to other parts of the year.

Overall results show that there is a large spread among the models, although the majority of the models simulate a similar seasonal variation. These differences suggest that despite the harmonization of inputs, such as emissions and boundary conditions, to regional models, there are still large differences between models, such as different gas-phase and aerosol modules, deposition schemes, meteorological drivers and spatial and vertical resolutions. Therefore, the use of multi-model ensembles can help to reduce the uncertainties inherent in individual models.

## Supplementary Material

Supp

## Figures and Tables

**Table 1.** Key features (meteorological/chemistry and transport models, emissions, horizontal and vertical grids) of the regional models participating to the AQMEII3 health impact study and the perturbation scenarios they performed.

Group code	Model	Emissions*	Horizontal resolution	Vertical resolution	Gas phase	Aerosol model	Europe				North America			

							BASE	GLO	NAM	EUR	BASE	GLO	EAS	NAM
DEI	COSMO-CLM/CMAQ	HTAP	24 km × 24 km	30 layers, 50hPa	CB5-TUCL	3 modes	×	×	×	×	×	×	×	×
DK1	WRF/DEHM	HTAP	17 km × 17 km	29 layers, 100 hPa	Brandt et al. (2012)	2 modes	×	×	×	×	×	×	×	×
ESI	WRF/CHEM	MACC	23 km × 23 km	33 layers, 50hPa	RADM2	3 modes, MADE/SORGAM	×		×					
FI1	ECMWF/SILAM	MACC+HTAP	0.25° × 0.25°	12 layers, 13 km	CB4	1–5 bins, VBS	×	×	×	×				
FRES1	ECMWF/CHIMERE	HTAP+HTAP	0.25° × 0.25°	9 layers, 50hPa	MELCHIOR2	8 bins	×	×	×	×				
IT1	WRF/CHEM	MACC	23 km × 23 km	33 layers, 50hPa	RACM-ESRL	3 modes, MADE/VBS	×	×		×				
IT2	WRF/CAMs	MACC	23 km × 23 km	14 layers, 8 km	CB5	3 modes	×	×						
NL1	LOTOS/EUROS	MACC	0.50° × 0.25°	4 layers, 3.5 km	CB4	2 modes, VBS	×							
TR1	WRF/CMAQ	MACC	30 km × 30 km	24 layers, lOhPa	CB5	3 modes	×	×	×					
UK1	WRF/CMAQ	MACC	15km × 15km	23 layers, 100 hPa	CB5-TUCL	3 modes	×	×	×	×				
UK2	WRF/CMAQ	HTAP	30 km × 30 km	23 layers, 100 hPa	CB5-TUCL	3 modes	×	×						
UK3	WRF/CMAQ	MACC	18km × 18km	35 layers, 16 km	CB5	3 modes	×	×	×					
US3	WRF/CMAQ	SMOKE	12km × 12km	35 layers, 50hPa	CB5-TUCL	3 modes					×	×	×	×

* MACC: modeling group used only the Monitoring Atmospheric Composition and Climate (MACC) emissions, MACC+HTAP: modeling group used MACC emissions for Europe and HTAP emissions over north Africa.

**Table 2.** Perturbations of global/regional anthropogenic emissions and boundary conditions in the perturbation scenarios.

	GLO	Europe		North America	

		NAM	EUR	NAM	EAS
Emissions	−20%	-	−20%	−20%	-
Boundary conditions (emissions in the IFS model)	−20%	20%	−20%	−20%	−20%

**Table 3.** Monthly statistics of Pearson’s correlation (r), normalized mean bias (NMB), normalized mean gross error (NMGE) and root mean square error (RMSE: pg m ^3^ for Europe, while ppb for gases and pg m^−3^ for particles for North America) calculated for each model group.

		Europe													North America						

		DEI	DK1	ESI	FI1	FRES1	IT1	IT2	TR1	UK1	UK2	Mean	Median	C-IFS	DEI	DK1	US1	US3	Mean	Median	C-IFS
O_3_	*r*	0.63	0.90	0.82	0.83	0.91	0.92	0.93	0.87	0.92	0.90	0.93	0.92	0.89	0.78	0.59	0.89	0.87	0.84	0.83	0.71
	NMB	0.10	0.07	−0.14	−0.36	−0.10	0.04	−0.14	0.09	0.08	−0.03	−0.04	−0.04	−0.20	0.12	0.22	0.14	−0.02	0.09	0.11	−0.10
	NMGE	0.17	0.12	0.15	0.36	0.12	0.13	0.15	0.26	0.11	0.09	0.08	0.08	0.20	0.17	0.23	0.14	0.08	0.12	0.13	0.19
	RMSE	12.68	8.81	11.58	23.13	9.01	8.54	10.94	17.66	8.05	6.79	5.91	6.31	14.63	6.16	9.81	5.72	3.23	4.63	5.28	7.31

NO_2_	*r*	0.80	0.88	0.89	0.95	0.74	0.90	0.92	0.90	0.85	0.85	0.95	0.93	0.92	0.99	0.92	0.94	0.93	0.98	0.99	0.91
	NMB	−0.75	−0.38	−0.47	0.00	0.05	−0.29	−0.30	0.58	−0.32	−0.06	−0.17	−0.24	0.07	−0.18	−0.35	0.05	0.31	−0.03	−0.02	0.41
	NMGE	0.75	0.38	0.47	0.20	0.23	0.29	0.30	0.58	0.32	0.17	0.18	0.24	0.20	0.18	0.35	0.10	0.31	0.06	0.02	0.41
	RMSE	9.38	5.41	6.00	2.89	3.44	4.43	4.15	7.39	4.65	2.74	2.70	3.49	2.59	1.01	2.05	0.62	1.77	0.40	0.26	2.30

CO	*r*	0.83	0.76	0.74	0.88	0.82	0.84	0.79	0.87	0.63	0.72	0.92	0.84	0.91	0.79	0.74	0.74	0.73	0.88	0.82	0.80
	NMB	−0.42	−0.42	−0.44	−0.27	−0.32	−0.38	−0.44	−0.20	−0.41	−0.43	−0.33	−0.38	−0.25	−0.19	−0.07	−0.06	−0.04	−0.07	−0.07	0.17
	NMGE	0.42	0.42	0.44	0.27	0.32	0.38	0.44	0.21	0.41	0.43	0.33	0.38	0.25	0.19	0.11	0.08	0.08	0.08	0.07	0.17
	RMSE	128.62	134.31	132.78	89.99	107.81	128.14	135.83	70.04	130.21	135.82	106.98	123.61	84.73	40.27	24.90	22.44	20.51	19.94	20.41	37.30

SO_2_	*r*	0.85	0.90	0.88	0.86	0.87	0.86	0.86	0.54	0.83	0.83	0.93	0.92	0.70	0.79	0.81	0.80	0.78	0.87	0.78	0.04
	NMB	−0.01	−0.47	−0.65	−0.20	−0.16	−0.30	−0.55	0.04	−0.13	0.20	−0.19	−0.10	0.41	−0.46	−0.42	0.07	−0.13	−0.19	−0.13	0.35
	NMGE	0.24	0.48	0.65	0.28	0.22	0.31	0.55	0.28	0.19	0.28	0.21	0.12	0.45	0.46	0.42	0.11	0.13	0.19	0.13	0.35
	RMSE	0.92	1.47	2.03	0.95	0.80	1.23	1.71	1.14	0.86	1.05	0.76	0.58	1.39	1.27	1.18	0.32	0.40	0.53	0.40	E02

PM_10_	*r*	0.86	0.82	0.17	0.41	0.82	0.60	0.10	0.52	0.71	0.71	0.87	0.73	−0.74	−0.31	−0.47	NA	0.07	0.47	−0.07	0.02
	NMB	−0.71	−0.59	−0.47	−0.42	−0.51	−0.20	−0.48	−0.25	−0.47	−0.42	−0.41	−0.45	−0.62	−0.67	−0.84	NA	−0.25	−0.44	−0.46	−0.86
	NMGE	0.71	0.59	0.47	0.42	0.51	0.25	0.48	0.26	0.47	0.42	0.41	0.45	0.62	0.67	0.84	NA	0.27	0.44	0.46	0.86
	RMSE	20.43	18.25	16.16	14.67	15.74	9.78	16.48	10.45	14.78	13.72	13.15	14.63	19.87	20.42	25.09	NA	9.85	13.51	14.74	25.58

PM_2.5_	*r*	0.89	0.86	0.24	0.58	0.84	0.75	0.11	0.62	0.77	0.77	0.89	0.82	−0.73	0.52	0.02	NA	0.54	0.61	0.56	0.18
	NMB	−0.64	−0.47	−0.27	−0.27	−0.36	−0.19	−0.48	−0.17	−0.40	−0.28	−0.32	−0.33	−0.59	−0.63	−0.14	NA	0.17	−0.15	−0.08	−0.39
	NMGE	0.64	0.47	0.35	0.30	0.36	0.24	0.49	0.24	0.41	0.30	0.32	0.33	0.59	0.63	0.20	NA	0.22	0.15	0.11	0.40
	RMSE	11.95	9.92	9.20	8.02	8.06	6.57	11.65	6.82	8.65	7.15	7.51	7.99	12.97	6.79	2.40	NA	2.78	1.92	1.41	5.04

NA - not available.

**Table 4.** Annual mean absolute differences (ppb for gases and pgm ^3^ for particles) between the base case and the different emission perturbation scenarios as calculated by the different model groups over the European domain.

Pollutant	Scenario	DE1	DK1	ES1	FI1	IT1	IT2	TR1	UK1	UK2	FRES1	All mean	Common mean
O_3_	GLO	−1.54	−0.71		−0.40	−0.37	−0.63	2.83	−0.83	−0.79	−0.63	−0.34	−0.82
	NAM	−0.28	−0.24	0.77	−0.13			−0.30	−0.22		−0.22	−0.09	−0.22
	EUR	−0.77	0.14		0.09	0.43			0.06		0.12	0.01	−0.07

NO_2_	GLO	−0.28	−0.72		−1.20	−0.93	−0.95	−1.93	−0.75	−1.10	−0.89	−0.97	−0.77
	NAM	0.00	0.01	0.17	0.00	0.00		0.01				0.03	0.00
	EUR	−0.30	−0.69		−1.05	−0.85			−0.70		−0.89	−0.75	−0.73

CO	GLO	−15.97	−14.03		−21.10	−18.13	−15.04	−26.01	−12.83	−16.94	−16.11	−17.35	−16.01
	NAM	−1.50	−1.71	3.26	−1.41			−1.35	−1.33		−1.55	−0.80	−1.50
	EUR	−10.49	−6.91		−14.63	−10.11			−7.87		−9.51	−9.92	−9.88

SO_2_	GLO	−0.23	−0.12		−0.17	−0.17	−0.11	−0.23	−0.20	−0.28	−0.15	−0.18	−0.17
	NAM	0.00	0.00	0.03	0.00			0.00	0.00		0.00	0.00	0.00
	EUR	−0.23	−0.10		−0.14	−0.13			−0.16		−0.15	−0.15	−0.16

PM_10_	GLO	−1.47	−1.90		−2.52	−2.97	−1.58	−3.58	−2.32	−2.81	−2.27	−2.38	−2.10
	NAM	−0.01	−0.09	0.00	−0.02			−0.04	−0.03		−0.04	−0.03	−0.04
	EUR	−2.03	−1.53		−2.20	−2.46			−1.96		−2.07	−2.04	−1.96

PM_2.5_	GLO	−1.30	−1.76		−2.15	−2.56	−1.33	−2.79	−1.78	−2.44	−2.10	−2.02	−1.82
	NAM	0.01	−0.05	0.00	−0.02			−0.03	−0.02		−0.04	−0.02	−0.02
	EUR	−1.29	−1.42		−1.82	−2.05			−1.47		−1.89	−1.66	−1.58

**Table 5.** Annual mean absolute differences (ppb for gases and pg m ^3^ for particles) between the base case and the different emission perturbation scenarios as calculated by the different model groups over the North American domain.

Pollutant	Scenario	DE1	DK1	US1	US3	All mean	Common mean
O_3_	GLO	−1.70	−1.42	−1.41	−1.03	−1.39	−1.39
	NAM	−0.92	−0.66		−0.36	−0.65	−0.65
	EAS	−0.35	−0.24	−0.23	−0.19	−0.25	−0.26

NO_2_	GLO	−0.35	−0.63	−1.07	−1.20	−0.81	−0.73
	NAM	−0.36	−0.62		−1.17	−0.71	−0.71
	EAS	0.00	0.00	0.00	−0.01	0.00	0.00

CO	GLO	−9.31	−20.48	−22.12	−25.01	−19.23	−18.27
	NAM	−3.84	−13.35		−19.87	−12.35	−12.35
	EAS	−2.60	−4.16	−3.64	−3.07	−3.37	−3.28

SO_2_	GLO	−0.33	−0.32	−0.48	−0.25	−0.34	−0.30
	NAM	−0.33	−0.32		−0.48	−0.37	−0.37
	EAS	0.00	0.00		0.00	0.00	0.00

PM_10_	GLO	−2.26	−0.66		−4.24	−2.39	−2.39
	NAM	−2.02	−0.59		−4.19	−2.27	−2.27
	EAS	−0.56	−0.05		−0.03	−0.21	−0.21

PM_2.5_	GLO	−0.60	−1.67		−2.29	−1.52	−1.52
	NAM	−0.62	−1.56		−2.24	−1.47	−1.47
	EAS	0.01	−0.04		−0.03	−0.02	−0.02

**Table 6.** Annual mean RERER values calculated for the multimodel mean (MMM) ensembles over Europe and North America.

	O_3_	NO_2_	CO	SO_2_	PM_10_	PM_2.5_
Europe						

DE1	0.44	−0.09	0.44	0.02	0.01	0.01
DK1	0.85	0.23	0.63	0.37	0.17	0.28
FI1	0.76	−0.01	0.40	0.01	0.02	0.02
FRES1	0.78	0.15	0.56	0.30	0.20	0.20
IT1	1.10	0.34	0.93	0.42	0.27	0.26
UK1	0.92	0.35	0.52	0.43	0.33	0.34
MMM	0.77	0.18	0.55	0.27	0.18	0.19
Median	0.81	0.19	0.54	0.34	0.18	0.23

North America						

DE1	0.77	0.12	0.73	0.07	0.09	0.12
DK1	0.93	0.06	0.90	0.15	0.07	0.12
US3	0.54	0.02	0.47	0.11	0.08	0.10
MMM	0.75	0.05	0.71	0.11	0.08	0.11
Median	0.77	0.06	0.73	0.11	0.08	0.12

## References

[R1] Appel KW, Pouliot GA, Simon H, Sarwar G, Pye HOT, Napelenok SL, Akhtar F, and Roselle SJ: Evaluation of dust and trace metal estimates from the Community Multiscale Air Quality (CMAQ) model version 5.0, Geosci. Model Dev, 6, 883–899, 10.5194/gmd-6-883-2013, 2013.

[R2] Akimoto H: Global air quality and pollution, Science, 302, 1716–1719, 10.1126/science.1092666, 2003. 14657488

[R3] Andersson E, Kahnert M, and Devasthale A: Methodology for evaluating lateral boundary conditions in the regional chemical transport model MATCH (v5.5.0) using combined satellite and ground-based observations, Geosci. Model Dev, 8, 3747–3763, 10.5194/gmd-8-3747-2015, 2015.

[R4] Brandt J, Silver JD, Frohn LM, Geels C, Gross A, Hansen AB, Hansen KM, Hedegaard GB, Skjøth CA, Villadsen H, Zare A, and Christensen JH: An integrated model study for Europe and North America using the Danish Eulerian Hemispheric Model with focus on intercontinental transport, Atmos. Environ, 53, 156–176, 10.1016/j.atmosenv.2012.01.011, 2012.

[R5] EMEP: Transboundary acidification, eutrophication and ground level ozone in Europe, Part I: Unified EMEP model description, EMEP status Report 1/2003, 2003.

[R6] Fiore A, Dentener F, Wild O, Cuvelier C, Schultz M, Textor C, Schulz M, Atherton C, Bergmann D, Bey I, Carmichael G, Doherty R, Duncan B, Faluvegi G, Folberth G, Garcia Vivanco M, Gauss M, Gong S, Hauglustaine D, Hess P, Holloway T, Horowitz L, Isaksen I, Jacob D, Jonson J, Kaminski J, keating T, Lupu A, MacKenzie I, Marmer E, Montanaro V, Park R, Pringle K, Pyle J, Sanderson M, Schroeder S, Shindell D, Stevenson D, Szopa S, Van Dingenen R, Wind P, Wojcik G, Wu S, Zeng G, and Zuber A: Multi-model estimates of intercontinental source-receptor relationships for ozone pollution, J. Geophys. Res, 114, D04301, 10.1029/2008JD010816, 2009.

[R7] Flemming J, Huijnen V, Arteta J, Bechtold P, Beljaars A, Blechschmidt A-M, Diamantakis M, Engelen RJ, Gaudel A, Inness A, Jones L, Josse B, Katragkou E, Marecal V, Peuch V-H, Richter A, Schultz MG, Stein O, and Tsikerdekis A: Tropospheric chemistry in the Integrated Forecasting System of ECMWF, Geosci. Model Dev, 8, 975–1003, 10.5194/gmd-8-975-2015, 2015.

[R8] Galmarini S, Bianconi R, Appel W, Solazzo E, Mosca S, Grossi P, Moran M, Schere K, and Rao ST: ENSEMBLE and AMET: Two systems and approaches to a harmonized, simplified and efficient facility for air quality models development and evaluation, Atmos. Environ, 53, 51–59, 2012.

[R9] Galmarini S, Kioutsioukis I, and Solazzo E: Epluribus unum*: ensemble air quality predictions, Atmos. Chem. Phys, 13, 7153–7182, 10.5194/acp-13-7153-2013, 2013.

[R10] Galmarini S, Koffi B, Solazzo E, Keating T, Hogrefe C, Schulz M, Benedictow A, Griesfeller JJ, Janssens-Maenhout G, Carmichael G, Fu J, and Dentener F: Technical note: Coordination and harmonization of the multi-scale, multi-model activities HTAP2, AQMEII3, and MICS-Asia3: simulations, emission inventories, boundary conditions, and model output formats, Atmos. Chem. Phys, 17, 1543–1555, 10.5194/acp-17-1543-2017, 2017. PMC5846500 29541091

[R11] Giordano L, Brunner D, Flemming J, Hogrefe C, Im U, Bianconi R, Badia A, Balzarini A, Baro R, Chemel C, Curci G, Forkel R, Jimenez-Guerrero P, Hirtl M, Hodzic A, Honzak L, Jorba O, Knote C, Kuenen JJP, Makar PA, Manders-Groot A, Neal L, Perez JL, Pirovano G, Pouliot G, San Jose R, Savage N, Schroder W, Sokhi RS, Syrakov D, To-rian A, Tuccella P, Werhahn J, Wolke R, Yahya K, Zabkar R, Zhang Y, and Galmarini S: Assessment of the MACC reanalysis and its influence as chemical boundary conditions for regional air quality modeling in AQMEII-2, Atmos. Environ, 115, 371–388, 2015.

[R12] Guenther AB, Jiang X, Heald CL, Sakulyanontvittaya T, Duhl T, Emmons LK, and Wang X: The Model of Emissions of Gases and Aerosols from Nature version 2.1 (MEGAN2.1): an extended and updated framework for modeling biogenic emissions, Geosci. Model Dev, 5, 1471–1492, 10.5194/gmd-5-1471-2012, 2012.

[R13] Hogrefe C, Liu P, Pouliot G, Mathur R, Roselle S, Flemming J, Lin M, and Park RJ: Impacts of different characterizations of large-scale background on simulated regional-scale ozone over the continental United States, Atmos. Chem. Phys, 18, 3839–3864, 10.5194/acp-18-3839-2018, 2018. 30079085 PMC6071430

[R14] Holloway T, Fiore A, and Hastings MG: Intercontinental transport of air pollution: Will emerging science lead to a new hemispheric treaty?, Environ. Sci. Technol, 37, 4535–4542, 10.1021/es034031g, 2003. 14594358

[R15] Huang M, Carmichael GR, Pierce RB, Jo DS, Park RJ, Flemming J, Emmons LK, Bowman KW, Henze DK, Davila Y, Sudo K, Jonson JE, Tronstad Lund M, Janssens-Maenhout G, Dentener FJ, Keating TJ, Oetjen H, and Payne VH: Impact of intercontinental pollution transport on North American ozone air pollution: an HTAP phase 2 multi-model study, Atmos. Chem. Phys, 17, 5721–5750, 10.5194/acp-17-5721-2017, 2017. 29780406 PMC5954439

[R16] Husar RB, Tratt DM, Schichtel DM, Falke SR, Li F, Jaffe D, Gassó S, Gill T, Laulainen NS, Lu F, Reheis MC, Chun Y, Westphal D, Holben BN, Gueymard C, McK-endry I, Kuring N, Feldman GC, McClain C, Frouin RJ, Merrill J, DuBois D, Vignola F, Murayama T, Nickovic S, Wilson WE, Sassen K, Sugimoto N, and Malm WC: Asian dust events of April 1998, J. Geophys. Res, 106, 18317–18330, 10.1029/2000JD900788, 2001.

[R17] Huszar P, Belda M, and Halenka T: On the long-term impact of emissions from central European cities on regional air quality, Atmos. Chem. Phys, 16, 1331–1352, 10.5194/acp-16-1331-2016, 2016.

[R18] Im U and Kanakidou M: Impacts of East Mediterranean megacity emissions on air quality, Atmos. Chem. Phys, 12, 6335–6355, 10.5194/acp-12-6335-2012, 2012.

[R19] Im U, Bianconi R, Solazzo E, Kioutsioukis I, Badia A, Balzarini A, Baro R, Belassio R, Brunner D, Chemel C, Curci G, Flemming J, Forkel R, Giordano L, Jimenez-Guerrero P, Hirtl M, Hodzic A, Honzak L, Jorba O, Knote C, Kuenen JJP, Makar PA, Manders-Groot A, Neal L, Perez JL, Piravano G, Pouliot G, San Jose R, Savage N, Schroder W, Sokhi RS, Syrakov D, Torian A, Werhahn K, Wolke R, Yahya K, Zabkar R, Zhang Y, Zhang J, Hogrefe C, and Galmarini S: Evaluation of operational online-coupled regional air quality models over Europe and North America in the context of AQMEII phase 2. Part I: Ozone, Atmos. Environ, 115, 404–420, 2015a.

[R20] Im U, Bianconi R, Solazzo E, Kioutsioukis I, Badia A, Balzarini A, Baro R, Bellasio R, Brunner D, Chemel C, Curci G, Denier van der Gon HAC, Flemming J, Forkel R, Giordano L, Jimenez-Guerrero P, Hirtl M, Hodzic A, Honzak L, Jorba O, Knote C, Makar PA, Manders-Groot A, Neal L, Perez JL, Pirovano G, Pouliot G, San Jose R, Savage N, Schroder W, Sokhi RS, Syrakov D, Torian A, Tuccella P, Werhahn K, Wolke R, Yahya K, Zabkar R, Zhang Y, Zhang J, Hogrefe C, and Galmarini S: Evaluation of operational online-coupled regional air quality models over Europe and North America in the context of AQMEII phase 2. Part II: Particulate Matter, Atmos. Environ, 115, 421–441, 2015b.

[R21] Im U, Brandt J, Geels C, Hansen KM, Christensen JH, Andersen MS, Solazzo E, Kioutsioukis I, Alyuz U, Balzarini A, Baro R, Bellasio R, Bianconi R, Bieser J, Colette A, Curci G, Farrow A, Flemming J, Fraser A, Jimenez-Guerrero P, Kitwiroon N, Liang C-K, Nopmong-col U, Pirovano G, Pozzoli L, Prank M, Rose R, Sokhi R, Tuccella P, Unal A, Vivanco MG, West J, Yarwood G, Hogrefe C, and Galmarini S: Assessment and economic valuation of air pollution impacts on human health over Europe and the United States as calculated by a multi-model ensemble in the framework of AQMEII3, Atmos. Chem. Phys, 18, 5967–5989, 10.5194/acp-18-5967-2018, 2018. 30079086 PMC6070159

[R22] Jaffe D, Bertschi I, Jaeglé L, Novelli P, Reid JS, Tanimoto H, Vingarzan R, and Westphal DL: Long-range transport of Siberian biomass burning emissions and impact on surface ozone in western North America, Geophys. Res. Lett, 31, L16106, 10.1029/2004GL020093, 2004.

[R23] Janssens-Maenhout G, Crippa M, Guizzardi D, Dentener F, Muntean M, Pouliot G, Keating T, Zhang Q, Kurokawa J, Wankmuller R, Denier van der Gon H, Kuenen JJP, Klimont Z, Frost G, Darras S, Koffi B, and Li M: HTAP_v2.2: a mosaic of regional and global emission grid maps for 2008 and 2010 to study hemispheric transport of air pollution, Atmos. Chem. Phys, 15, 11411–11432, 10.5194/acp-15-11411-2015, 2015.

[R24] Jiménez P, Parra R, and Baldasano JM: Influence of initial and boundary conditions for ozone modeling in very complex terrains: a case study in the northeastern Iberian Peninsula, Environ. Modell. Softw, 22, 1294–1306, 2007.

[R25] Jonson JE, Schulz M, Emmons L, Flemming J, Henze D, Sudo K, Tronstad Lund M, Lin M, Benedictow A, Koffi B, Dentener F, Keating T, and Kivi R: The effects of intercontinental emission sources on European air pollution levels, Atmos. Chem. Phys. Discuss, 10.5194/acp-2018-79, in review, 2018.

[R26] Kioutsioukis I, Im U, Solazzo E, Bianconi R, Badia A, Balzarini A, Bard R, Bellasio R, Brunner D, Chemel C, Curci G, van der Gon HD, Flemming J, Forkel R, Giordano L, Jiménez-Guerrero P, Hirtl M, Jorba O, Manders-Groot A, Neal L, Pérez JL, Pirovano G, San Jose R, Savage N, Schroder W, Sokhi RS, Syrakov D, Tuccella P, Werhahn J, Wolke R, Hogrefe C, and Galmarini S: Insights into the deterministic skill of air quality ensembles from the analysis of AQMEII data, Atmos. Chem. Phys, 16, 15629–15652, 10.5194/acp-16-15629-2016, 2016.

[R27] Li Q, Jacob DJ, Bey I, Palmer PI, Duncan BN, Field BD, Martin RV, Fiore AM, Yantosca RM, Parrish DD, Simmonds PG, and Oltmans SJ: Transatlantic transport of pollution and its effects on surface ozone in Europe and North America, J. Geophys. Res, 107, 4166, 10.1029/2001JD001422, 2002.

[R28] Liang C-K, West JJ, Silva RA, Bian H, Chin M, Den-tener FJ, Davila Y, Emmons L, Folberth G, Flemming J, Henze D, Im U, Jonson JE, Kucsera T, Keating TJ, Lund MT, Lenzen A, Lin M, Pierce RB, Park RJ, Pan X, Sekiya T, Sudo K, and Takemura T: HTAP2 multimodel estimates of premature human mortality due to intercontinental transport of air pollution, Atmos. Chem. Phys. Discuss, 10.5194/acp-2017-1221, in review, 2018. PMC7668558 33204242

[R29] Mason R, Zubrow A, and Eyth A: Technical Support Document (TSD) Preparation of Emissions Inventories for the Version 5.0, 2007 Emissions Modeling Platform, available at:https://www.epa.gov/air-emissions-modeling/ 2007-version-50-technical-support-document (last access: 25 June 2018), 2012.

[R30] Mathur R: Estimating the impact of the 2004 Alaskan forest fires on episodic particulate matter pollution over the eastern United States through assimilation of satellite-derived aerosol optical depths in a regional air quality model, J. Geophys. Res, 113, D17302, 10.1029/2007JD009767, 2008.

[R31] Pouliot G, Denier van der Gon H, Kuenen J, Makar P, Zhang J, and Moran M: Analysis of the emission inventories and model-ready emission datasets of Europe and North America for phase 2 of the AQMEII project, Atmos. Environ, 115, 345–360, 2015.

[R32] Rao S, Mathur R, Hogrefe C Keating T, Dentener F, and Galmarini S: Path Forward for the Air Quality Model Evaluation International Initiative (AQMEII), EM, Air And Waste Management Associations Magazine For Environmental Managers, 7, 38–41, 2012.

[R33] Rudich Y, Kaufman YJ, Dayan U, Yu H, and Kleidman RG: Estimation of transboundary transport of pollution aerosols by remote sensing in the eastern Mediterranean, J. Geophys. Res, 113, D14S13, 10.1029/2007JD009601, 2008.

[R34] Solazzo E, Bianconi R, Vautard R, Appel KW, Moran MD, Hogrefe C, Bessagnet B, Brandt J, Christensen JH, Chemel C, Coll I, van der Gon HD, Ferreira J, Forkel R, Francis XV, Grell G, Grossi P, Hansen AB, Jerice-vic A, Kraljevic L, Miranda AI, Nopmongcol U, Pirovano G, Prank M, Riccio A, Sartelet KN, Schaap M, Silver JD, Sokhi RS, Vira J, Werhahn J, Wolke R, Yarwood G, Zhang J, Rao ST, and Galmarini S: Ensemble modelling of surface level ozone in Europe and North America in the context of AQMEI, Atmos. Environ, 53, 60–74, 2012a.

[R35] Solazzo E, Bianconi R, Pirovano G, Matthias V, Vautard R, Moran MD, Appel KW, Bessagnet B, Brandt J, Christensen JH, Chemel C, Coll I, Ferreira J, Forkel R, Francis XV, Grell G, Grossi P, Hansen AB, Hogrefe C, Miranda AI, Nopmongco U, Prank M, Sartelet KN, Schaap M, Silver JD, Sokhi RS, Vira J, Werhahn J, Wolke R, Yarwood G, Zhang J, Rao ST, and Galmarini S: Operational model evaluation for particulate matter in Europe and North America in the context of AQMEII, Atmos. Environ, 53, 75–92, 2012b.

[R36] Solazzo E, Riccio A, Kioutsioukis I, and Galmarini S: Pauci ex tanto numero: reduce redundancy in multi-model ensembles, Atmos. Chem. Phys, 13, 8315–8333, 10.5194/acp-13-8315-2013, 2013.

[R37] Solazzo E, Hogrefe C, Colette A, Garcia-Vivanco M, and Galmarini S: Advanced error diagnostics of the CMAQ and Chimere modelling systems within the AQMEII3 model evaluation framework, Atmos. Chem. Phys, 17, 10435–10465, 10.5194/acp-17-10435-2017, 2017a. PMC6104839 30147711

[R38] Solazzo E, Bianconi R, Hogrefe C, Curci G, Tuccella P, Alyuz U, Balzarini A, Bard R, Bellasio R, Bieser J, Brandt J, Christensen JH, Colette A, Francis X, Fraser A, Vi-vanco MG, Jiménez-Guerrero P, Im U, Manders A, Nop-mongcol U, Kitwiroon N, Pirovano G, Pozzoli L, Prank M, Sokhi RS, Unal A, Yarwood G, and Galmarini S: Evaluation and error apportionment of an ensemble of atmospheric chemistry transport modeling systems: multivariable temporal and spatial breakdown, Atmos. Chem. Phys, 17, 3001–3054, 10.5194/acp-17-3001-2017, 2017b. PMC6105295 30147713

[R39] Song C-K, Byun DW, Pierce RB, Alsaadi JA, Schaack TK, and Vukovich F: Downscale linkage of global model output for regional chemical transport modeling: method and general performance, J. Geophys. Res, 113, D08308, 10.1029/2007JD008951, 2008.

[R40] Stjern CW, Samset BH, Myhre G, Bian H, Chin M, Davila Y, Dentener F, Emmons L, Flemming J, Haslerud AS, Henze D, Jonson JE, Kucsera T, Lund MT, Schulz M, Sudo K, Takemura T, and Tilmes S: Global and regional radiative forcing from 20 % reductions in BC, OC and SO4 - an HTAP2 multi-model study, Atmos. Chem. Phys, 16, 13579–13599, 10.5194/acp-16-13579-2016, 2016.

[R41] Tang Y, Carmichael GR, Thongboonchoo N, Chai T, Horowitz LW, Pierce RB, Al-Saadi JA, Pfister G, Vukovich JM, Avery MA, Sachse GW, Ryerson TB, Holloway JS, Atlas EL, Flocke FM, Weber RJ, Huey G, Dibb JE, Streets DG, and Brune WH: Influence of lateral and top boundary conditions on regional air quality prediction: a multiscale study coupling regional and global chemical transport models, J. Geophys. Res, 112, D10S18, 10.1029/2006JD007515, 2007.

[R42] Tuccella P, Curci G, Grell GA, Visconti G, Crumeyrolle S, Schwarzenboeck A, and Mensah AA: A new chemistry option in WRF-Chem v. 3.4 for the simulation of direct and indirect aerosol effects using VBS: evaluation against IMPACT-EUCAARI data, Geosci. Model Dev, 8, 2749–2776, 10.5194/gmd-8-2749-2015, 2015.

[R43] UN (United Nations): Hemispheric transport of air pollution 2007. Air Pollution Studies No. 16, Interim report prepared by the Task Force on Hemispheric Transport of Air Pollution acting within the framework of the Convention on Long-range Transboundary Air Pollution, New York and Geneva, 2007.

[R44] Vivanco MG, Theobald MR, García-Gómez H, Garrido JL, Prank M, Aas W, Adani M, Alyuz U, Andersson C, Bellasio R, Bessagnet B, Bianconi R, Bieser J, Brandt J, Brig-anti G, Cappelletti A, Curci G, Christensen JH, Colette A, Couvidat F, Cuvelier K, D’Isidoro M, Flemming J, Fraser A, Geels C, Hansen KM, Hogrefe C, Im U, Jorba O, Kitwiroon N, Manders A, Mircea M, Otero N, Pay M-T, Pozzoli L, Solazzo E, Tsyro S, Unal A, Wind P, and Gal-marini S: Modelled deposition of nitrogen and sulfur in Europe estimated by 14 air quality model-systems: Evaluation, effects of changes in emissions and implications for habitat protection, Atmos. Chem. Phys. Discuss, 10.5194/acp-2018-104, in review, 2018. PMC6235743 30450115

[R45] Wilkening KE, Barrie LA, and Engle M: Atmospheric science: Trans-Pacific air pollution, Science, 290, 65–67, 10.1126/science.290.5489.65, 2000. 11183151

[R46] World Health Organization (WHO): Review of evidence on health aspects of air pollution (REVIHAAP), WHO Technical Report, Copenhagen, Denmark, 2013.

