# Supplementary material for: Influence of anthropogenic emissions and boundary conditions on multi-model simulations of major air pollutants over Europe and North America in the framework of AQMEII3
Source: Atmos Chem Phys. Author manuscript; Available in PMC 2018 Aug 22. (PMC6104647; doi:10.5194/acp-18-8929-2018)
Supplement: Supp [file NIHMS982662-supplement-Supp.pdf]

Supplement of Atmos. Chem. Phys., 18, 8929–8952, 2018
https://doi.org/10.5194/acp-18-8929-2018-supplement
© Author(s) 2018. This work is distributed under
the Creative Commons Attribution 4.0 License.

Atmospheric Chemistry and Physics
Open Access

Supplement of

Influence of anthropogenic emissions and boundary conditions on multi-model simulations of major air pollutants over Europe and North America in the framework of AQMEII3

Ulas Im et al.

Correspondence to: Ulas Im (ulas@envs.au.dk)

The copyright of individual parts of the supplement might differ from the CC BY 4.0 License.

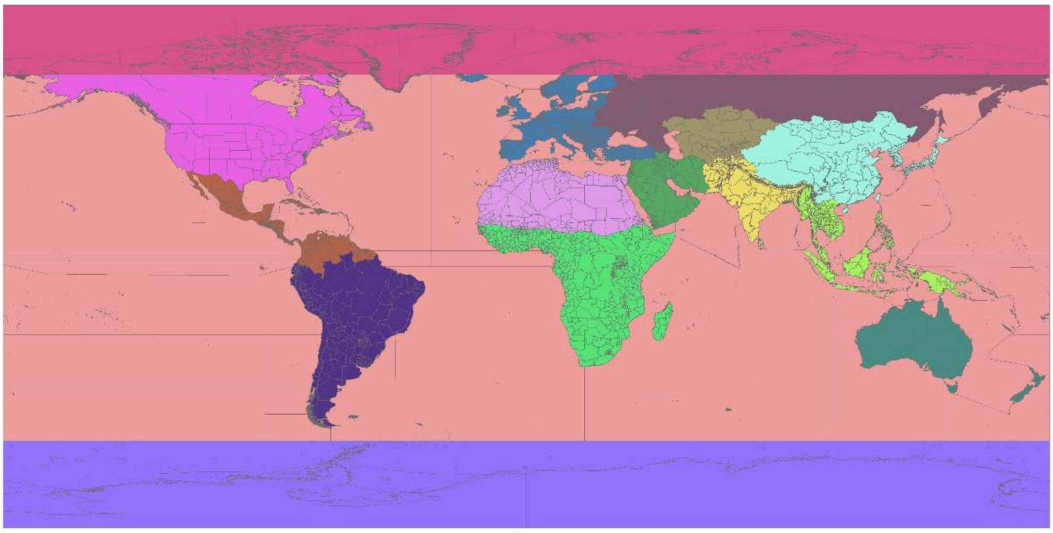

Fig. S1. The HTAP2 regions of source/receptor areas (Taken from Galmarini et al. (2017))

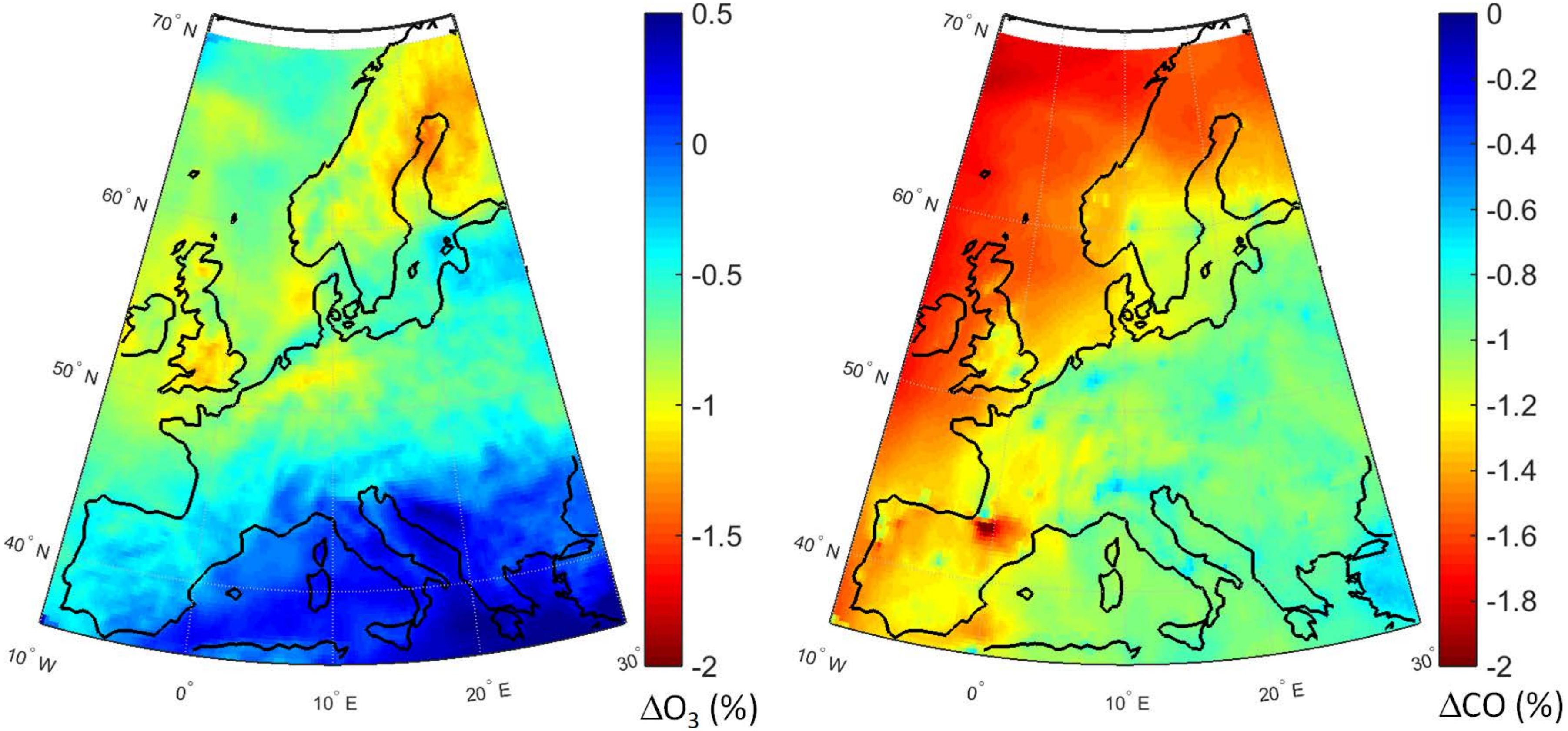

Fig. S2. Response of European springtime O_3 and CO levels to the 20% reduction if the North American anthropogenic emissions (NAM)

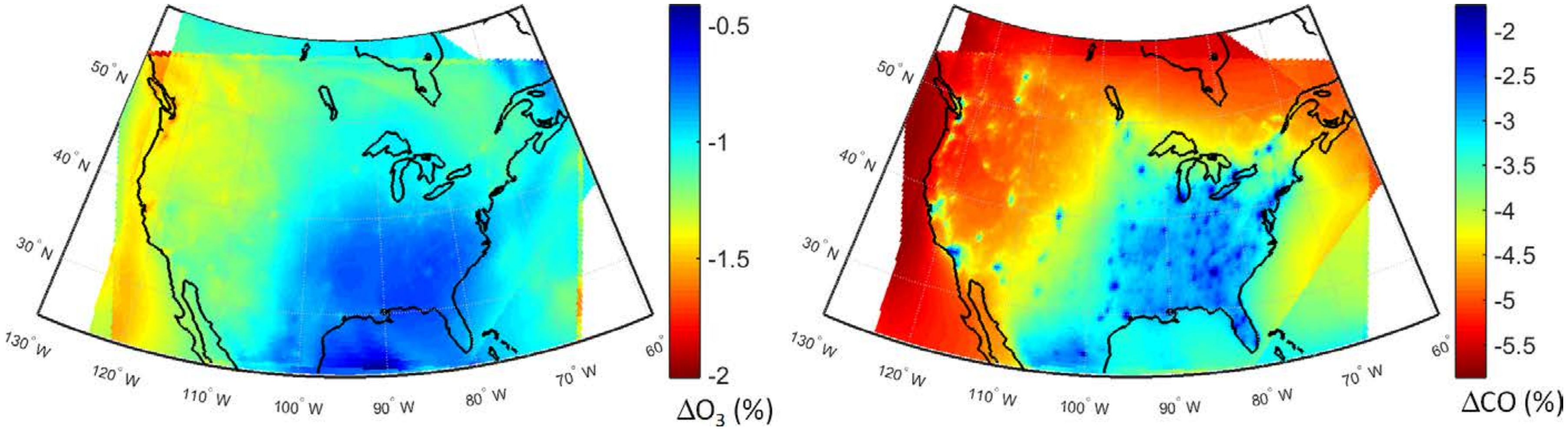

Fig. S3. Response of European springtime O_3 and CO levels to the 20% reduction in the East Asian anthropogenic emissions (EAS).
